# Calculated Serum Antioxidant Capacity Masks Opposite Associations of Urate and Unconjugated Bilirubin with Leukocyte Count: A Ten-Year Cross-Sectional Hospital Laboratory Study Including 25-Hydroxyvitamin D

**DOI:** 10.3390/ijms27219483

**Published:** 2026-10-23

**Authors:** Joško Osredkar, Uroš Godnov, Aleš Jerin, Darko Siuka

**Affiliations:** 1 Clinical Institute of Clinical Chemistry and Biochemistry, University Medical Centre Ljubljana, Zaloška Cesta 2, 1000 Ljubljana, Slovenia; josko.osredkar@kclj.si (J.O.); ales.jerin@kclj.si (A.J.); 2 Faculty of Pharmacy, University of Ljubljana, Aškerčeva Cesta 7, 1000 Ljubljana, Slovenia; 3 Faculty of Mathematics, Natural Sciences and Information Technologies, University of Primorska, Glagoljaška Ulica 8, 6000 Koper, Slovenia; uros.godnov@upr.si; 4 Department of Gastroenterology, University Medical Centre Ljubljana, Zaloška Cesta 2, 1000 Ljubljana, Slovenia; 5 Faculty of Medicine, University of Ljubljana, Vrazov trg 2, 1000 Ljubljana, Slovenia

**Keywords:** total antioxidant capacity, uric acid, bilirubin, leukocytes, inflammation, oxidative stress, 25-hydroxyvitamin D, albumin, biomarkers

## Abstract

Calculated total antioxidant capacity (cTAC), a weighted sum of albumin, urate and bilirubin, is often interpreted as a single antioxidant dimension. We asked whether its constituents share the same association with leukocyte count, a routine but nonspecific marker of the inflammatory, oxidant-generating cell compartment, and examined 25-hydroxyvitamin D [25(OH)D]. In this retrospective cross-sectional study of routine records from a tertiary university hospital laboratory (2014–2023; 20,525 patient-years, 14,152 adults), cTAC was calculated with Trolox-equivalent coefficients (0.63·albumin + 1.02·urate + 1.50·bilirubin). Patient-clustered models of log leukocyte count, leukopenia (<4 × 10^9^/L) and leukocytosis (≥10 × 10^9^/L) were adjusted for age, sex, season and era, and additionally for estimated glomerular filtration rate and alanine aminotransferase. Urate accounted for 90% of the variance of cTAC. cTAC showed a U-shaped association with leukocytosis, whereas its components diverged. Per standard deviation, urate was associated with more leukocytes (+4.2%, 95% CI 3.2–5.2; leukocytosis odds ratio [OR] 1.23, 1.14–1.32) and calculated unconjugated bilirubin with fewer (−4.3%, −5.7 to −2.9; OR 0.71, 0.63–0.82). Both associations were similar after multiple imputation of missing covariates. Platelet adjustment attenuated the bilirubin estimate to −1.4%, whereas haemoglobin adjustment did not; with platelet counts of 150–400 × 10^9^/L and no anaemia, it was −3.8% (−5.0 to −2.7). 25(OH)D was weakly inversely associated (−1.2%, −1.8 to −0.6), without evidence of effect modification. In this cross-sectional setting, in which residual confounding is likely, a single calculated antioxidant capacity statistically conceals opposite-signed associations of its constituents with leukocyte count.

## 1. Introduction

Oxidative stress denotes an imbalance between the generation of oxidants and antioxidant defence in favour of oxidants, leading to disrupted redox signalling and molecular damage [1]. Because individual reactive species are short-lived and difficult to quantify in clinical samples, laboratory medicine has long relied on integrated measures of the antioxidant side of this balance. The Trolox-equivalent antioxidant capacity (TEAC) assay developed by Miller, Rice-Evans and colleagues ranked the endogenous plasma antioxidants [2]. Albumin and urate accounted for most of the activity of plasma, with smaller contributions from ascorbate, α-tocopherol and bilirubin. Other assays, such as the ferric-reducing ability of plasma (FRAP), likewise attribute the largest share to urate [3]. Measured total antioxidant capacity (TAC) and indices calculated from its constituents have since been applied to the study of antioxidant status in many clinical settings [4,5]. At the same time, it has been repeatedly cautioned that TAC values are assay-dependent composites whose interpretation is ambiguous when their constituents change for reasons unrelated to redox status [6,7]. In this paper, TAC denotes a measured assay result, TEAC the assay and its compound-specific coefficients, and cTAC the calculated index derived from routine analytes; cTAC is not a measurement of antioxidant capacity.

This ambiguity is sharpest for the three dominant endogenous contributors, each of which has a biology that extends far beyond radical scavenging. Urate scavenges peroxyl radicals and singlet oxygen in the extracellular compartment [8], but within cells it can behave as a pro-oxidant and pro-inflammatory molecule [9]. Its formation by xanthine oxidoreductase is itself a source of superoxide and hydrogen peroxide [10]; monosodium urate crystals activate the NLRP3 inflammasome [11], and urate released from injured cells acts as an endogenous danger signal [12]. Serum urate is accordingly associated with leukocyte count and other inflammatory markers in population studies [13,14], and it is strongly determined by kidney function [15]. Unconjugated bilirubin, the end product of haem degradation by haem oxygenase-1 (HO-1) and biliverdin reductase, is a lipophilic chain-breaking antioxidant at physiological concentrations [16]; a biliverdin–bilirubin redox cycle has been proposed to amplify this activity [17], although its quantitative contribution has been questioned [18]. Beyond scavenging, bilirubin inhibits leukocyte migration and NADPH oxidase activity [19,20], and the HO-1 axis is broadly anti-inflammatory [21]. Mildly elevated bilirubin, including Gilbert’s syndrome, has been associated with lower inflammatory burden and cardiovascular risk [22,23], and serum bilirubin has been inversely associated with leukocyte count and C-reactive protein in adults [24,25]. Albumin, the largest contributor to plasma TEAC through its free thiol and metal-binding sites [26], is also a negative acute-phase protein whose concentration falls with inflammation [27]. Vitamin D, finally, is not a direct radical scavenger but regulates antioxidant and immune gene programmes, including Nrf2-dependent responses [28,29,30].

The leukocyte count provides a natural reference against which these components can be compared. Activated neutrophils and monocytes are the main circulating source of reactive oxygen and chlorine species through NADPH oxidase and myeloperoxidase [31], and the leukocyte count is a widely available, prognostically informative, although nonspecific, marker of systemic inflammation [32]. If cTAC captured a single protective antioxidant dimension, higher cTAC and each of its components would be expected to be concordantly associated with lower leukocyte counts. If, instead, its constituents carry distinct and partly inflammatory signals, a composite index would average opposite associations and obscure them. Previous studies have examined urate and bilirubin one at a time in relation to inflammatory markers [13,24], or have examined measured TAC as a single exposure [5]; how the opposing associations of the constituents combine within a calculated index has not, to our knowledge, been examined. Composite oxidative balance scores are also increasingly used in epidemiology [33], which makes this question of general methodological relevance.

We therefore used ten years of routine laboratory data from a tertiary university hospital to decompose cTAC into its albumin, urate and bilirubin contributions, and to compare the composite and its components in relation to leukocyte count, leukopenia and leukocytosis. We also contrasted calculated unconjugated bilirubin with measured direct bilirubin, which shares analytical and hepatic determinants. Finally, we examined whether serum 25(OH)D is associated with leukocyte count and modifies these associations.

## 2. Results

### 2.1. Study Population

The laboratory extract contained 107,900 records with a 25(OH)D request between 2014 and 2023. Of these, 107,856 had a valid 25(OH)D result, sampling date, sex and date of birth, and 82,235 belonged to adults aged 18–100 years. The largest reduction followed from the requirement that leukocyte count, albumin, urate, and total and direct bilirubin be reported on the same request and within plausibility ranges (24,811 records), because urate and direct bilirubin were not part of most vitamin D requests. Retaining the first record per patient and calendar year left 20,527 records, and 20,525 patient-years from 14,152 individuals had complete base covariates (Table S1). Most patients (10,356; 73.2%) contributed a single patient-year, and 49.5% of patient-years came from patients with two or more. The median age was 63.0 years (IQR 46.0–74.8) and 56.7% were women. The median 25(OH)D was 59 nmol/L (IQR 38–79); 15.9% had 25(OH)D < 30 nmol/L. The median leukocyte count was 7.0 × 10^9^/L (IQR 5.7–8.6); 3.8% had leukopenia and 12.4% leukocytosis (Table 1). Compared with patient-years with normal counts, those with leukopenia had lower albumin, urate and haemoglobin and higher unconjugated bilirubin, whereas those with leukocytosis had higher urate and lower unconjugated bilirubin. eGFR was missing in 0.1% and ALT in 35.0% of patient-years (Table S8a).

### 2.2. Composition of Calculated Antioxidant Capacity

**Figure 1:**
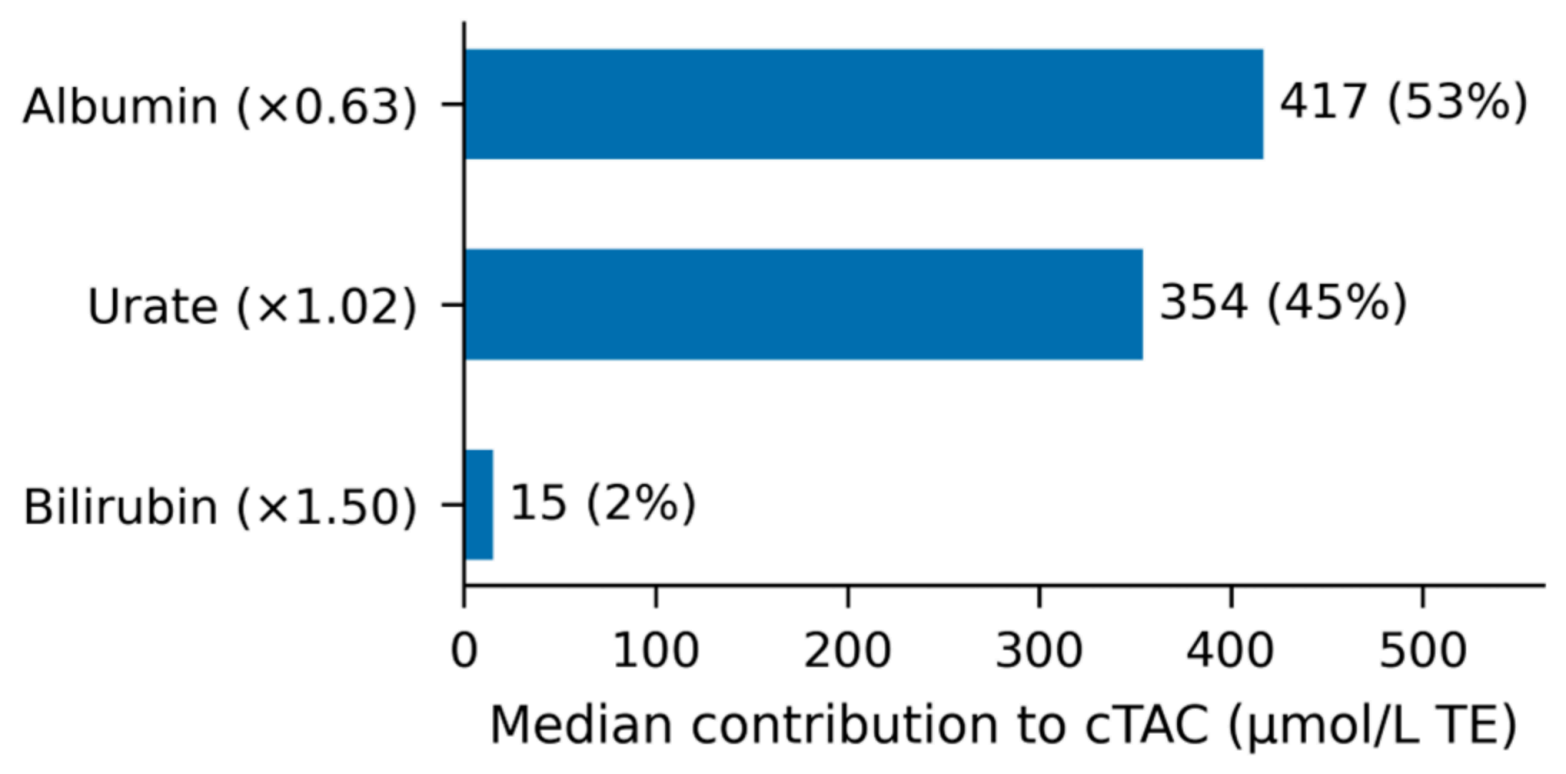
Median weighted albumin, urate and total-bilirubin contributions to calculated serum antioxidant capacity (cTAC; µmol/L Trolox equivalents, TE). Parentheses show each component median as a percentage of the sum of the three component medians; these differ slightly from the medians of the patient-year percentage contributions in Table S2. TEAC coefficients are given in the labels.

The median cTAC was 786 µmol/L Trolox equivalents (IQR 706–873). Albumin contributed a median 417 µmol/L (52.5% of cTAC), urate 354 µmol/L (45.2%) and bilirubin 15 µmol/L (1.9%) (Figure 1, Table S2). Although albumin provided the largest share of the cTAC level, urate accounted for 90.5% of its variance across patient-years (r = 0.92), albumin for 7.1% and bilirubin for 2.4% (Table S2). We also calculated the index with FRAP stoichiometric weights, as an unweighted molar sum, and with each coefficient decreased or increased by 50%. Across these schemes, urate accounted for 63–97% of the variance of the index, and each index remained positively associated with leukocyte count (Table S10a). 25(OH)D correlated positively with albumin (ρ = 0.167) and unconjugated bilirubin (ρ = 0.073) but inversely with urate (ρ = −0.095), so that its correlation with the composite was close to zero (ρ = −0.034; Table S3).

### 2.3. The Composite Index and Leukocyte Count

Per standard deviation (SD; 128 µmol/L), cTAC was associated with a 2.9% higher leukocyte count (95% CI 2.3–3.5), i.e., in the direction opposite to that expected for a protective antioxidant pool (Table 2, model A). Higher cTAC was associated with lower odds of leukopenia (OR 0.74, 95% CI 0.66–0.82) but slightly higher odds of leukocytosis (OR 1.07, 1.01–1.13). Across cTAC quintiles, the prevalence of leukocytosis was 15.2%, 10.6%, 10.0%, 12.0% and 14.2%, and that of leukopenia fell from 5.7% to 2.0–3.1% (Table S4), suggesting a U-shaped association with leukocytosis. A model using urate in place of cTAC was associated with a larger difference in leukocyte count (+3.6% per SD, 2.9–4.3) and explained slightly more of its variation (R^2^ 1.9% vs. 1.6%) (Table S10b).

### 2.4. Decomposition: Urate and Unconjugated Bilirubin Diverge

When the components were entered simultaneously (Table 2, model C), urate was associated with a higher leukocyte count (+3.5% per SD, 95% CI 2.8–4.1) and calculated unconjugated bilirubin with a lower count (−4.6% per SD, −5.8 to −3.3). The two components also had mirror-image associations with the tails of the distribution. Urate was associated with more leukocytosis (OR 1.19, 1.13–1.25) and less leukopenia (OR 0.85, 0.76–0.94), and unconjugated bilirubin with less leukocytosis (OR 0.71, 0.64–0.79) and more leukopenia (OR 1.19, 1.08–1.31). Albumin was inversely associated with both leukopenia (OR 0.60, 0.57–0.65) and leukocytosis (OR 0.84, 0.80–0.88), consistent with its role as a global marker of health status rather than a directional correlate of leukocyte number. Direct bilirubin showed no clear association with leukocyte count (+1.1%, −0.1 to 2.2) or leukopenia (OR 0.96, 0.89–1.03) and only a weak positive association with leukocytosis (OR 1.09, 1.00–1.19). Collinearity was low (variance inflation factors ≤ 1.9).

**Figure 2:**
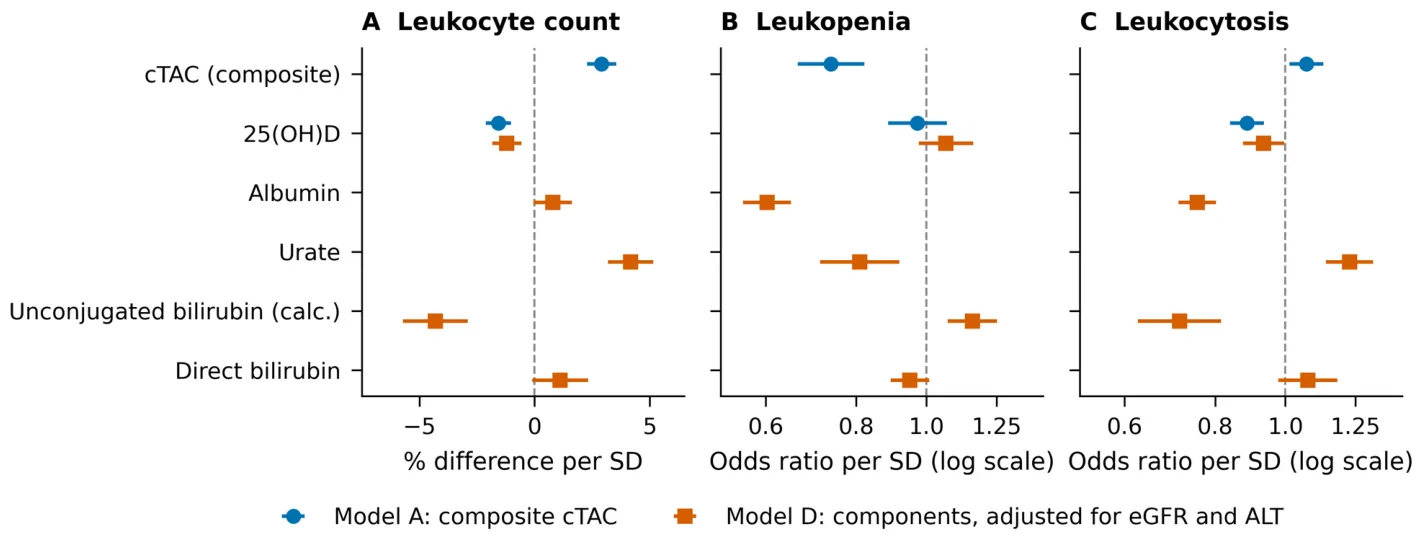
Composite versus decomposed antioxidant capacity. Estimates per SD of each predictor for (**A**) leukocyte count (percentage difference) and for (**B**) leukopenia (<4 × 10^9^/L) and (**C**) leukocytosis (≥10 × 10^9^/L) (odds ratios, log scale). Blue circles, model A (composite cTAC; n = 20,525 patient-years); orange squares, model D (components, adjusted for log eGFR and log ALT; n = 13,320). Bars, patient-clustered 95% confidence intervals.

**Figure 3:**
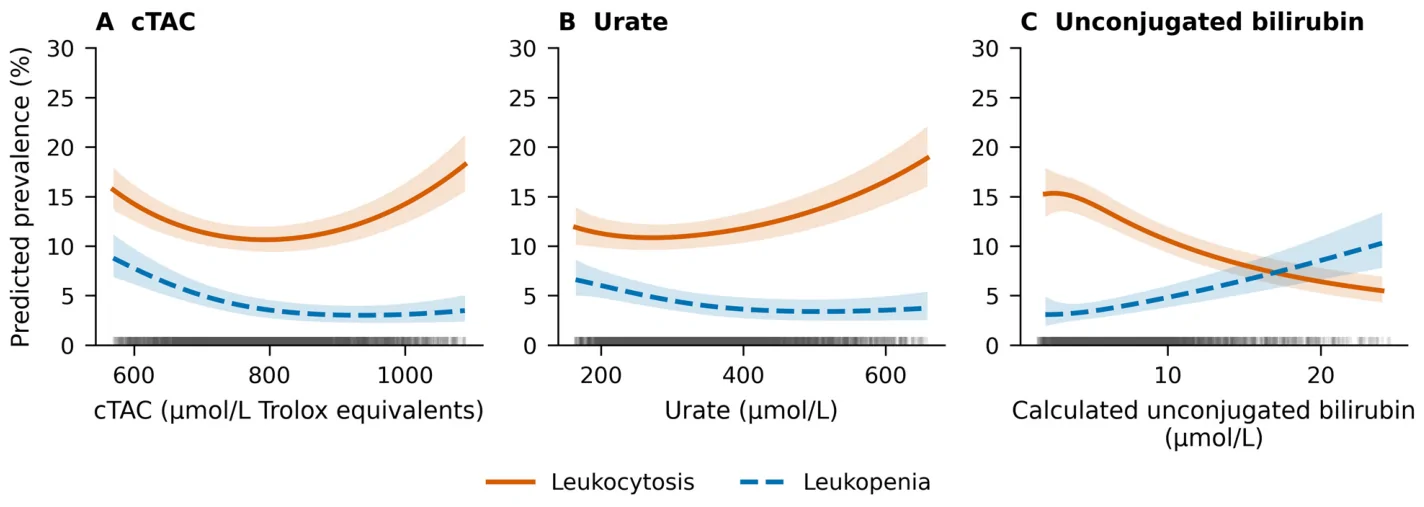
Predicted prevalence of leukocytosis (solid orange line) and leukopenia (dashed blue line) across (**A**) cTAC, (**B**) urate and (**C**) calculated unconjugated bilirubin, from logistic models with cubic B-splines (4 df), shown between the 2nd and 98th percentiles. Shaded bands, patient-clustered 95% confidence intervals; tick marks, values of a random sample of 3000 patient-years. Models were fitted in 20,525 patient-years and adjusted for age, sex, season, era and 25(OH)D; panels B and C additionally included the other components (albumin, urate or calculated unconjugated bilirubin, direct bilirubin). Reference profile: woman, 60 years, winter, modal era, mean 25(OH)D and mean values of the other components.

Additional adjustment for eGFR and ALT (model D; n = 13,320 patient-years with eGFR and ALT) did not materially change these estimates: urate +4.2% (3.2–5.2) per SD and leukocytosis OR 1.23 (1.14–1.32); unconjugated bilirubin −4.3% (−5.7 to −2.9) and leukocytosis OR 0.71 (0.63–0.82) (Figure 2). In clinical units, these estimates correspond to +3.4% (2.6–4.2) per 100 µmol/L urate and −2.8% (−3.8 to −1.9) per 5 µmol/L unconjugated bilirubin; at the median leukocyte count, one SD corresponds to about 0.3 × 10^9^/L (Table S9a). Estimates of model C refitted in the same subset were similar (Table S5b), and a multinomial model with normal counts as the reference category gave relative risk ratios almost identical to the binary odds ratios (Table S9b). Predicted leukocytosis showed a U-shaped relationship with cTAC (Figure 3A). In B-spline models mutually adjusted for the other components, predicted leukocytosis increased with urate above approximately 300 µmol/L and generally decreased with unconjugated bilirubin over the displayed range (Figure 3B,C). In the same subset, R^2^ for log leukocyte count was 1.4% for the covariates alone, 2.0% for model A, 3.8% for model C and 4.3% for model D. These models therefore explained only a small proportion of the variation in log leukocyte count.

### 2.5. Missing Covariate Data

Patient-years without ALT (35.0%) were older, had lower eGFR and higher urate, and came mostly from the period before 2020 (Table S8b). We therefore fitted model D without ALT in 20,508 patient-years and after multiple imputation in all 20,525 patient-years. Estimates were similar to, or slightly stronger than, the complete-case estimates. After imputation, urate was associated with +4.3% (95% CI 3.6–5.0) per SD and a leukocytosis OR of 1.25 (1.18–1.32), and unconjugated bilirubin with −4.9% (−6.3 to −3.5) and an OR of 0.67 (0.60–0.76) (Table S8c).

### 2.6. Haematological, Hepatic and Nutritional Covariates

Because haemoglobin and platelet count share haem-turnover and marrow determinants with bilirubin and leukocytes, we examined them separately (Table 3). Haemoglobin adjustment did not attenuate the unconjugated bilirubin estimate (−4.7% per SD, 95% CI −6.3 to −3.0). Platelet adjustment attenuated it to −1.4% (−2.1 to −0.7). Joint adjustment gave −1.8% (−2.7 to −0.8), with a leukocytosis OR of 0.89 (0.79–1.00) and a leukopenia OR of 0.97 (0.90–1.04). Platelet count was positively correlated with leukocyte count (ρ = 0.37) and inversely with unconjugated bilirubin (ρ = −0.19). Further adjustment for mean corpuscular volume and red cell distribution width did not change these estimates, and excluding anaemia alone did not attenuate the bilirubin estimate (−5.4%, −6.6 to −4.1). The subset with platelet counts of 150–400 × 10^9^/L and no anaemia included 9094 patient-years. In it, the bilirubin estimate was −3.8% (−5.0 to −2.7), with a leukocytosis OR of 0.76 (0.64–0.89), and it remained −3.6% (−4.7 to −2.5) after adjustment for haemoglobin and platelets. There was no clear association with leukopenia in this subset (OR 1.06, 0.88–1.29). Adjustment for log γ-glutamyltransferase and log alkaline phosphatase, or for total protein and glucose, did not change the bilirubin or urate estimates. The urate association was similar or stronger in every model in Table 3 (+4.0% to +5.0% per SD). Albumin changed sign after adjustment for haemoglobin or platelets (−1.9% with either), indicating that its direction depends on the adjustment set.

### 2.7. 25-Hydroxyvitamin D

In model D, 25(OH)D was weakly inversely associated with leukocyte count (−1.2% per SD of 31.5 nmol/L, 95% CI −1.8 to −0.6) and with slightly lower odds of leukocytosis (OR 0.93, 0.87–1.00); there was no clear association with leukopenia in that model (OR 1.06, 0.98–1.16). The inverse association with leukocyte count was present in all models (Table 2). There was no evidence that 25(OH)D modified the association of the composite (interaction −0.1% per SD of each factor, −0.6 to 0.5; *p* = 0.7) or of urate (0.3%, −0.3 to 1.0; *p* = 0.3) with leukocyte count. The interaction estimate for unconjugated bilirubin was 0.0% (−1.2 to 1.1; *p* = 0.9) (Table S5a, models B and F). These confidence intervals include no interaction and small interactions, but not interactions larger than about 1% per SD of each factor.

### 2.8. Sensitivity Analyses

Urate and unconjugated bilirubin retained opposite associations in every sensitivity set (Table 4). These included one record per patient; exclusion of leukocyte counts ≥ 30 × 10^9^/L, eGFR < 30 mL/min/1.73 m^2^, albumin < 35 g/L or total bilirubin > 34 µmol/L; women and men separately; and the periods before and after 2020. Per-SD estimates ranged from +2.6% to +5.5% for urate and from −2.4% to −5.5% for unconjugated bilirubin; the smallest bilirubin estimates were observed after exclusion of total bilirubin > 34 µmol/L and within the normal leukocyte range (4 to <10 × 10^9^/L; linear model). The one-record-per-patient analysis, which kept each patient’s first eligible record of the whole 2014–2023 period (9391 patients with eGFR and ALT), gave +3.3% (2.3–4.4) for urate and −4.7% (−5.8 to −3.5) for unconjugated bilirubin. Estimates were similar in patients with a single patient-year and in those with repeated patient-years (interaction *p* = 0.8 for both components), and after weighting each patient-year by the inverse of the patient’s number of patient-years. Replacing calendar era by calendar-year fixed effects, standardising each analyte within calendar year, or modelling age with 3 or 6 spline degrees of freedom or linearly did not change the estimates (Table 4 and Table S9c). After restriction to direct bilirubin > 2 µmol/L, the bilirubin estimate was −3.2% (−4.4 to −2.0), with a leukocytosis OR of 0.82 (0.72–0.93) (Table S5b); restriction to >3 µmol/L gave −2.6% (−3.7 to −1.5) (Table S11a). These restrictions exclude the lowest reported direct-bilirubin values but also change the sample.

## 3. Discussion

In more than 20,000 patient-years of routine laboratory data, calculated serum antioxidant capacity was not associated with leukocyte count in the way expected of a protective antioxidant pool. The composite showed a U-shaped association with leukocytosis, while its components had opposite associations. Urate, which supplies almost half of cTAC and 90% of its variance, was associated with more leukocytes. Calculated unconjugated bilirubin was associated with fewer leukocytes; the bilirubin term supplies only about 2% of cTAC. Because cTAC varies almost entirely with urate, the inverse bilirubin association is not visible in the composite. In this sense the composite “masks” the bilirubin association statistically; the term does not imply a biological mechanism. Direct bilirubin, which shares analytical, hepatic and sampling determinants with the calculated unconjugated fraction, showed no clear association with leukocyte count in the primary models. 25(OH)D showed a small inverse association and no evidence of effect modification. All of these are cross-sectional associations, and the mechanisms discussed below are hypotheses that the data cannot test.

### 3.1. Urate and Leukocyte Count: Competing Explanations

The positive association of urate with leukocyte count agrees with population studies linking serum urate to leukocyte count and inflammatory markers [13,14]. Its direction cannot be determined from cross-sectional data, and at least three explanations are compatible with it. First, leukocyte number may raise urate: urate is the end product of purine catabolism, and increased cell production and turnover increase urate formation, most dramatically in tumour lysis [34]. Second, urate may contribute to inflammation. Extracellular urate is a major plasma scavenger of oxidants [8]. However, its production by xanthine oxidoreductase generates superoxide and hydrogen peroxide [10], and intracellular urate can promote oxidative stress and NADPH oxidase activation [9]. Monosodium urate crystals activate the NLRP3 inflammasome and IL-1β release, which promotes neutrophil recruitment [11]. Soluble urate, without crystal formation, has also been reported to prime cytokine production by human mononuclear cells and to activate the NLRP3 inflammasome in experimental models [35,36]. Third, urate and leukocytes may share determinants that we could not measure, such as obesity and the metabolic syndrome, reduced renal clearance, diuretic use or acute illness. The association persisted after adjustment for eGFR, the principal determinant of serum urate [15], after excluding observations with eGFR < 30 mL/min/1.73 m^2^, and after adjustment for haematological, hepatic and nutritional covariates, and it was the most consistent finding of the study. This dual chemistry, antioxidant in plasma but associated with oxidant-generating and pro-inflammatory pathways, is the main reason why a urate-dominated index cannot be read as a measure of antioxidant protection.

### 3.2. Unconjugated Bilirubin: Haematological and Analytical Considerations

The inverse association of unconjugated bilirubin with leukocyte count is consistent with experimental findings that bilirubin inhibits leukocyte adhesion and transendothelial migration and suppresses NADPH oxidase [19,20]. Related evidence concerns the anti-inflammatory HO-1/biliverdin reductase pathway [21] and lower inflammatory markers in individuals with mildly elevated bilirubin [22,23]. Tsai et al. reported an inverse association between total bilirubin and leukocyte count in an adult population [24]. Our hospital analysis examines calculated unconjugated and measured direct bilirubin separately and places bilirubin within a composite antioxidant index. These studies, however, do not establish the mechanism of the association we observed, and our data cannot show that bilirubin itself lowers the leukocyte count.

The haematological analyses (Table 3) show where the association is sensitive. Haemoglobin, a marker of erythrocyte mass and haem supply, did not attenuate the association, and neither did excluding anaemia, whereas platelet count did. Thrombocytopenia and leukopenia can coexist in cirrhosis and portal hypertension [37], and marrow suppression or ineffective erythropoiesis can also lower blood counts. Such clinical states are possible shared determinants of low platelet and leukocyte counts and of bilirubin, but diagnoses, splenic status and direct markers of haemolysis or ineffective erythropoiesis were unavailable, so their contribution cannot be established here. The bilirubin estimate was therefore sensitive to platelet adjustment, which is consistent with shared haematological determinants, with correlated markers or with adjustment-related bias; these possibilities cannot be distinguished cross-sectionally. Platelet count can also be a co-marker of inflammation (reactive thrombocytosis). Whether it is a confounder, a mediator or a collider depends on causal relationships that this study cannot establish. We therefore present model D without and with the additional haematological covariates, alongside the restricted analyses (Table 3). With platelet counts of 150–400 × 10^9^/L and no anaemia, the association with leukopenia was no longer clear. The inverse association with leukocyte count (−3.8% per SD) and leukocytosis persisted and changed little after adjustment for haemoglobin and platelets. Restriction does not, however, remove residual confounding. An exploratory comparison of 77 patient-years with isolated unconjugated hyperbilirubinaemia, a Gilbert-like phenotype, with 5489 patient-years with normal bilirubin showed no difference in leukocyte count (+0.3%, 95% CI −6.2 to 7.2; Table S11b). This comparison was imprecise and did not provide evidence of an inverse association for this phenotype.

The unconjugated fraction was not measured but calculated as total minus direct bilirubin, and direct bilirubin assays also detect albumin-bound conjugated (δ) bilirubin [38]. The calculated value is therefore an estimate of indirect bilirubin, not an analytical measurement of unconjugated bilirubin, and measured direct bilirubin is an operational measurement rather than a pure conjugated fraction. Bilirubin was reported in whole µmol/L, and 36.8% of direct-bilirubin values were ≤2 µmol/L, where this resolution and assay imprecision are large relative to the value. The models with total and direct bilirubin and with calculated unconjugated and direct bilirubin gave the same estimate (−4.3% per SD of the calculated fraction; Table S11a); this is expected from the reparameterisation and does not validate measurement accuracy. Classical, independent measurement error in a predictor attenuates its association towards the null [39]. Here, however, both assays contribute error, and errors shared with the direct-bilirubin covariate can affect multivariable estimates in either direction, so the direction and magnitude of any bias cannot be determined. Restriction to direct bilirubin > 2 or >3 µmol/L weakened, but did not remove, the association (−3.2% and −2.6%); these restrictions also change the distribution of direct bilirubin and the composition of the sample, and they do not quantify measurement error. The difference between direct and calculated unconjugated bilirubin shows that the two have different conditional associations with leukocyte count; it does not establish biological specificity or an antioxidant or anti-inflammatory effect.

### 3.3. Albumin and 25(OH)D

Albumin was inversely associated with both leukopenia and leukocytosis. This is the pattern of a nonspecific marker of health status: albumin falls in acute and chronic inflammation [27,40] and in the malnutrition and catabolic states that accompany cytopenias. Its small positive association with leukocyte count in model D was reversed after adjustment for haemoglobin or platelets, indicating that its direction depends on the adjustment set. Albumin supplies more than half of the cTAC level, largely through Cys34 and metal binding [26], but because its concentration varies little relative to that of urate, it contributed only 7% of the variance of cTAC. Across patient-years, cTAC therefore behaves essentially as a transformed urate concentration (r = 0.92), which explains why the composite says little about how any single antioxidant is associated with the inflammatory leukocyte compartment.

The inverse association of 25(OH)D with leukocyte count was small (about 1% per SD, i.e., less than 0.1 × 10^9^/L) and of uncertain biological significance. It is consistent with the immunomodulatory actions of vitamin D [28,30], but equally with the lower 25(OH)D of acutely ill, less mobile patients, with seasonal and supplementation patterns that were not recorded, and with the indications for 25(OH)D testing, which were unknown. It should not be read as evidence that vitamin D reduces systemic inflammation. We found no statistical evidence of interaction of 25(OH)D with the composite or with urate or unconjugated bilirubin. Absence of evidence is not evidence of absence: the confidence intervals exclude large interactions but not small ones, and they do not support the hypothesis that vitamin D and endogenous antioxidants act synergistically on the leukocyte compartment.

### 3.4. Implications for Oxidative-Stress Biomarkers

The practical message concerns the interpretation of TAC and related composite indices [4,6,33]. Because albumin and urate dominate both calculated and measured non-enzymatic antioxidant capacity [2,3], a single TAC value integrates a negative acute-phase protein, a purine metabolite with pro-inflammatory associations, and a haem-derived molecule with anti-inflammatory properties. In our data these constituents were associated with leukocyte count in different directions. The U-shaped association of cTAC with leukocytosis may reflect these differing component associations, such as low albumin in acutely ill patients at low cTAC and high urate at high cTAC. Case mix and residual confounding are other possibilities, and its origin was not established. The log-count model estimates an average multiplicative association, whereas the leukocytosis model estimates the probability of exceeding a fixed threshold; these summaries need not have the same shape, so the positive linear association and the U-shape are not contradictory. No clinically actionable threshold was established, and we do not consider the U-shape a clinically meaningful finding.

The effects were modest. One SD of urate or unconjugated bilirubin corresponded to about 0.3 × 10^9^/L, and the main models explained less than 5% of the variation in log leukocyte count. We did not evaluate patient-level prediction of inflammation, and these results do not establish clinical predictive utility or validate a new biomarker. The contribution of the study is methodological: a composite antioxidant index can conceal associations of its constituents that operate in opposite directions, and for the present outcome cTAC explained slightly less variation in log leukocyte count than urate alone in otherwise comparable models. We therefore propose, as a hypothesis for further evaluation rather than an established recommendation, that studies reporting measured TAC or calculated cTAC, or the related antioxidant gap, also report albumin, urate and bilirubin and analyse them separately. Whether the same masking occurs for measured TAC, for other inflammatory markers such as C-reactive protein, and in other populations needs to be tested.

### 3.5. Strengths and Limitations

Strengths include the large number of patient-years, the availability of all index analytes and the leukocyte count on the same laboratory request, and patient-clustered inference. Other strengths are the use of published TEAC coefficients and alternative weights, the contrast between calculated unconjugated and direct bilirubin, and extensive sensitivity analyses. The study also has important limitations.

First, it is cross-sectional and observational, so no causal direction can be inferred, and residual confounding is likely. The extract contained no diagnoses, comorbidities, smoking status, medications, and C-reactive protein or leukocyte differential count. The total leukocyte count cannot distinguish neutrophilic changes from changes in other leukocyte populations; it is a nonspecific proxy for systemic inflammation and does not measure oxidant generation. Leukopenia and leukocytosis were defined by fixed thresholds that do not identify the clinical causes of abnormal counts. Adjustment for eGFR, ALT and other routine analytes cannot remove confounding by unmeasured disease. The E-values were approximately 1.5 for urate and 1.6 for unconjugated bilirubin [41], i.e., an unmeasured confounder associated with both the analyte and leukocytosis by risk ratios of this size could explain the point estimates. Infection, malignancy or acute illness could plausibly produce confounding of this strength in a hospital population.

Second, treatments may have influenced the associations in several directions. Xanthine oxidase inhibitors lower urate and xanthine-oxidase-derived oxidative stress [42], and their users may also differ in underlying disease. Diuretics raise urate. Glucocorticoids raise neutrophil counts through marrow release and demargination [43]. Cytotoxic chemotherapy lowers leukocytes, while cell lysis can raise urate unless urate-lowering prophylaxis prevents it [34]. Because the direction of these effects differs by drug class, their net influence cannot be predicted, and prospective studies with information on therapeutic class are needed.

Third, the cohort is a selected stream of hospital and outpatient requests in which vitamin D and a broad chemistry panel were co-requested. Such patients are older and more often ill than the general population, and the associations, and especially the U-shaped pattern, may differ in healthy populations. Patients tested in several years contributed several patient-years; their number of tests may reflect disease severity, but estimates were similar in patients with single and repeated patient-years and in the one-record-per-patient analysis.

Fourth, cTAC is a calculated proxy and not a measured capacity. The TEAC coefficients were obtained for purified compounds in one assay [2] and may not hold in plasma, in disease, or in other assays; ascorbate, tocopherol and non-albumin thiols, which contribute to measured TAC, were not available. Our main conclusion, however, did not depend on the coefficients (Table S10a). We could not quantify low-concentration bilirubin measurement error or reconstruct the effects of historical assay changes, and measurement error in direct bilirubin propagates into the calculated unconjugated fraction. Calendar-year adjustment and within-year standardisation addressed systematic drift but not changes in precision. Finally, ALT was missing in about one-third of patient-years, mainly before 2020. Multiple imputation gave similar results, but it relies on the assumption that missingness depended only on the observed variables, and the imputation model ignored the clustering of patient-years within patients.

### 3.6. Relation to Companion Analyses

The same laboratory infrastructure and period underlie a separate analysis of 25(OH)D and serum urate [44]. That study concerns the vitamin D–urate association; the present analysis concerns the leukocyte count as an outcome and the decomposition of a composite antioxidant index, in which urate is one of three components. No results are duplicated.

## 4. Materials and Methods

### 4.1. Data Source and Ethics

This retrospective cross-sectional study used anonymised routine laboratory records from the Clinical Institute of Clinical Chemistry and Biochemistry, University Medical Centre Ljubljana, Slovenia, from 1 January 2014 to 31 December 2023. The Institute is the central laboratory of the country’s largest tertiary hospital and receives inpatient, outpatient and community requests; the extract did not contain care-setting labels, diagnoses or ordering indications. The extract included all records with a serum 25(OH)D request together with the chemistry and haematology results reported on the same request. The study was approved by the Republic of Slovenia National Medical Ethics Committee (KSEV-5-111125, 11 November 2025) for secondary analysis of previously collected, anonymised data; informed consent was waived. Personal identifiers were replaced by a one-way cryptographic hash before analysis. The study is reported according to STROBE [45] (Table S6).

### 4.2. Cohort

The cohort rule was identical to that used in companion analyses of the same extract and was fixed before the present analysis. Eligible records required a valid 25(OH)D result, sampling date, sex and date of birth, and age 18–100 years. They also required leukocyte count, albumin, urate, total bilirubin and direct bilirubin on the same request, within prespecified physiological plausibility ranges (Table S7b). Values outside those ranges were set to missing, and records missing an index analyte were excluded. To limit the influence of frequently tested patients while preserving seasonal and calendar-period information, the first record per patient per calendar year was retained (patient-year). Using all records would give greater weight to patients tested repeatedly within a year, typically during the same illness episode. Using one record per patient would discard subsequent annual observations. The primary analysis therefore retained one record per patient-year and used patient-clustered standard errors; a sensitivity analysis retained each patient’s first eligible record of 2014–2023.

### 4.3. Laboratory Measurements and Derived Variables

All analytes were measured in the Institute’s routine laboratory on the platforms in clinical service during the period, under continuous internal quality control and external quality assessment. Serum 25(OH)D was measured by electrochemiluminescence immunoassay (Elecsys Vitamin D Total, Roche Diagnostics, Mannheim, Germany). Albumin, urate, total and direct bilirubin, creatinine, ALT, γ-glutamyltransferase, alkaline phosphatase, total protein and glucose were measured by routine photometric methods on the Siemens ADVIA clinical chemistry platform (Roche Diagnostics GmbH, Mannheim, Germany). Leukocyte and platelet counts, haemoglobin concentration, mean corpuscular volume and red cell distribution width were measured on automated haematology analysers (Siemens ADVIA 1800, Erlangen, Sysmex Europe SE, Hamburg, Germany). Because platforms and reagent lots changed over the decade, calendar era was included in all models, and calendar-year fixed effects and within-year standardisation of the analytes were examined in sensitivity analyses. Bilirubin results were reported in whole µmol/L. Historical assay-specific limits of quantification and concentration-dependent precision data were not available in the extract; the reporting resolution describes the stored values and does not establish analytical accuracy at low concentrations. Unconjugated (indirect) bilirubin was calculated as total minus direct bilirubin (negative values set to 0, which occurred once); direct bilirubin is an operational assay measurement and is not equivalent to a pure conjugated fraction. eGFR was calculated with the 2021 race-free CKD-EPI creatinine equation. Seasons were defined meteorologically, and calendar era as 2014–2019 versus 2020–2023. Anaemia was defined as haemoglobin < 120 g/L in women and <130 g/L in men.

Calculated serum antioxidant capacity (µmol/L Trolox equivalents) was defined from the TEAC values of the individual antioxidants reported by Miller et al. [2]:

cTAC = 0.63 × albumin (µmol/L) + 1.02 × urate (µmol/L) + 1.50 × total bilirubin (µmol/L)

with albumin converted from g/L to µmol/L using a molecular mass of 66.5 kDa. These coefficients are the molar antioxidant activities of the purified compounds relative to Trolox in the TEAC assay. We used them because they were derived in the study that established the relative contributions of the plasma antioxidants [2] and because all three analytes are routinely measured. Total bilirubin was used, as in the original coefficients. As sensitivity analyses, we recalculated the index with FRAP stoichiometric factors (urate 2.0, bilirubin 4.0; albumin set to 0 because its FRAP activity is very low [3]), as an unweighted molar sum, with calculated unconjugated instead of total bilirubin, and with each TEAC coefficient decreased or increased by 50%. Ascorbate and tocopherol, which contribute about 12% of plasma TEAC [2], were not available. A non-albumin cTAC (urate and bilirubin terms only) was analysed secondarily because albumin is a negative acute-phase protein. The contribution of each component to the variance of cTAC across analytic patient-years was computed as cov (component term, cTAC)/var (cTAC).

### 4.4. Outcomes

The primary outcome was the natural logarithm of leukocyte count; coefficients were back-transformed to percentage differences per SD of the predictor. Secondary outcomes were leukopenia (<4 × 10^9^/L) and leukocytosis (≥10 × 10^9^/L), the conventional limits of the adult reference interval. Separate binary logistic models compared each tail with all remaining patient-years, because each was a state of interest. The reference group therefore included the opposite abnormal tail: normal counts comprised 95.6% of non-leukocytosis and 87.1% of non-leukopenia patient-years. A multinomial logistic model with normal counts (4 to <10 × 10^9^/L) alone as the reference category was fitted as a sensitivity analysis.

### 4.5. Statistical Analysis

25(OH)D, cTAC and the component analytes were standardised to mean 0 and SD 1 across all patient-years. The SDs were 31.5 nmol/L for 25(OH)D, 128 µmol/L for cTAC, 4.9 g/L for albumin, 123.1 µmol/L for urate, 7.7 µmol/L for calculated unconjugated bilirubin and 10.9 µmol/L for direct bilirubin; log eGFR and log ALT were not standardised. Estimates for leukocyte count are percentage differences and those for leukopenia and leukocytosis odds ratios, each with 95% confidence intervals (CIs). All models included a B-spline of age (4 df), sex, season and era (base covariates). Model A added 25(OH)D and cTAC to the base covariates, and model B added their interaction. Model C included 25(OH)D and all components (albumin, urate, calculated unconjugated and direct bilirubin), and model D additionally log eGFR and log ALT. Model E included 25(OH)D, non-albumin cTAC, their interaction, albumin, log eGFR and log ALT. Model F added interactions of 25(OH)D with urate and with calculated unconjugated bilirubin to model D (specifications in Table S7a). Inference used cluster-robust standard errors by patient. All splines were cubic B-splines with 4 df (one internal knot at the median, boundary knots at the observed range); this specification was fixed in advance and was not selected by model fit. Age with 3 or 6 df and linear age were examined as sensitivity analyses. Logistic models with B-splines of cTAC, urate or calculated unconjugated bilirubin examined non-linearity; the urate and bilirubin models also included the other components. Predicted probabilities with patient-clustered 95% CI were computed for a woman aged 60 years, in winter and the modal era, with mean 25(OH)D and mean values of the other components. Collinearity was assessed with variance inflation factors. Pairwise associations were summarised with Spearman correlations and the cTAC–urate association with Pearson’s r. Variance explained was compared as in-sample R^2^ of linear models fitted to identical samples. Clinical-unit estimates were obtained by entering the predictors per stated unit, and absolute differences were evaluated at the median leukocyte count.

Sensitivity analyses repeated model D using one record per patient and separately in women and men and before and after 2020. Further restrictions excluded leukocyte counts ≥ 30 × 10^9^/L, total bilirubin > 34 µmol/L, eGFR < 30 mL/min/1.73 m^2^ or albumin < 35 g/L; these cutoffs reduce the influence of marked haematological or biochemical abnormalities but do not identify or exclude specific diagnoses. Model D was also restricted to leukocyte counts of 4 to < 10 × 10^9^/L (linear model only), to direct bilirubin > 2 and >3 µmol/L, and to red cell distribution width ≤ 15%, and it was refitted with total bilirubin in place of calculated unconjugated bilirubin, retaining direct bilirubin and expressing the coefficient per SD of the calculated fraction. Repeated testing was defined by the number of patient-years per patient in the full cohort. We fitted model D separately in patients with single and repeated patient-years. Repeated-testing interactions were fitted separately for urate and calculated unconjugated bilirubin by adding a repeated-testing indicator (at least two patient-years) and its product with the standardised analyte to the log-count model D. We also weighted each patient-year by the inverse of the patient’s number of patient-years (linear model only). We also replaced era by calendar-year fixed effects and standardised the analytes within calendar year.

We added haemoglobin, log platelet count, mean corpuscular volume and red cell distribution width to model D; further analyses added log γ-glutamyltransferase and log alkaline phosphatase, or total protein and glucose. Each comparison refitted model D in the same available-data subset. Model D was also repeated after excluding anaemia and in patient-years with platelet counts of 150–400 × 10^9^/L and without anaemia. An exploratory analysis compared patient-years with isolated unconjugated hyperbilirubinaemia (total bilirubin > 17 µmol/L with ≥80% calculated unconjugated) with those with total bilirubin ≤ 17 µmol/L. Both groups required direct bilirubin ≤ 5 µmol/L, no anaemia, red cell distribution width ≤ 15%, ALT ≤ 35 U/L (women) or ≤45 U/L (men) and platelet counts of 150–400 × 10^9^/L. Models were adjusted for the base covariates, 25(OH)D, albumin, urate and log eGFR; eight otherwise eligible reference patient-years without eGFR were excluded.

The primary analysis used complete cases for ALT and eGFR. As sensitivity analyses, we fitted model D without ALT and after multiple imputation by chained equations (statsmodels MICEData) with predictive mean matching (10 donors), five burn-in cycles before the first and two cycles between subsequent imputations, and 20 imputed data sets. The imputation model contained log leukocyte count, the standardised analytes, log eGFR, log ALT, log aspartate aminotransferase, log γ-glutamyltransferase, haemoglobin, log platelet count, age (linear), sex, season and era, and it did not model the clustering of patient-years within patients; missing auxiliary values were imputed within the same procedure (random seed 20261018), and no convergence diagnostics are reported. Each imputed data set was analysed with model D and patient-clustered standard errors, and estimates were combined by Rubin’s rules [46]. E-values were computed for the leukocytosis odds ratios after conversion to approximate risk ratios (square-root transformation for a common outcome) [41]. Because estimation, not hypothesis testing, was the aim, results are reported as point estimates with 95% CI; Wald *p*-values are given only for interaction terms. Analyses were performed in Python 3.13 (pandas, statsmodels, SciPy, matplotlib); the code is available from the corresponding author. A generative AI tool (Claude, Anthropic) assisted in writing the analysis code; all code and outputs were checked by the authors (see Acknowledgments).

## 5. Conclusions

Calculated serum antioxidant capacity is dominated in level by albumin and urate and in its variation by urate, and in this hospital population it explained slightly less variation in log leukocyte count than urate alone. Its constituents were associated with leukocyte count in opposite directions: urate positively and consistently, and calculated unconjugated bilirubin inversely. The inverse bilirubin estimate attenuated after platelet adjustment and persisted in the subset with platelet counts of 150–400 × 10^9^/L and no anaemia. 25(OH)D showed a small inverse association and no evidence of effect modification. These cross-sectional associations do not establish that urate promotes, or bilirubin protects against, inflammation. They show, however, that a single composite antioxidant value can conceal opposite associations of its components with a marker of the inflammatory cell compartment. Studies of antioxidant capacity should therefore report and interpret the composite together with its components, a proposal that should next be tested with measured antioxidant capacity and specific inflammatory markers.

## Figures and Tables

**Table 1:** Characteristics of the study population by leukocyte category.

Characteristic	All	Leukopenia (<4 × 10^9^/L)	Normal (4–<10 × 10^9^/L)	Leukocytosis (≥10 × 10^9^/L)
Patient-years, n	20,525	783	17,197	2545
Age, years	63.0 [46.0–74.8]	60.0 [46.7–69.7]	63.4 [46.8–75.0]	62.2 [39.8–74.6]
25(OH)D, nmol/L	59 [38–79]	55 [33–80]	59 [39–79]	55 [34–79]
Leukocytes, ×10^9^/L	7.0 [5.7–8.6]	3.5 [3.1–3.8]	6.8 [5.7–7.9]	11.3 [10.5–12.7]
Albumin, g/L	44 [41–46]	42 [37–45]	44 [41–46]	43 [40–46]
Urate, µmol/L	347 [270–434]	316 [248–410]	347 [272–433]	355 [267–451]
Total bilirubin, µmol/L	10 [7–14]	12 [8–20]	10 [7–14]	9 [6–12]
Unconjugated bilirubin (calculated), µmol/L	7 [5–10]	8 [5–12]	7 [5–10]	6 [4–8]
Direct bilirubin, µmol/L	3 [2–4]	4 [3–8]	3 [2–4]	3 2–4[]
cTAC, µmol/L TE	786 [706–873]	744 [675–836]	787 [711–872]	788 [690–885]
eGFR, mL/min/1.73 m^2^	76.3 [41.5–100.2]	83.1 [41.0–103.9]	76.4 [42.2–99.5]	73.1 [36.9–105.8]
ALT, U/L	24.0 [18.0–34.2]	24.6 [18.0–37.2]	24.6 [18.0–33.6]	22.8 [16.8–34.2]
Haemoglobin, g/L	133 [120–144]	121 [107–133]	133 [121–144]	132 [119–144]
Women, %	56.7	58.1	56.5	57.4

Values are median [interquartile range] unless stated otherwise. Models requiring eGFR and ALT included 13,320 patient-years. eGFR, CKD-EPI 2021 creatinine equation. 25(OH)D, 25-hydroxyvitamin D; ALT, alanine aminotransferase; cTAC, calculated total antioxidant capacity; eGFR, estimated glomerular filtration rate; TE, Trolox equivalents.

**Table 2:** Composite and decomposed antioxidant capacity and leukocyte outcomes, per SD of each predictor.

Model	Predictor (Per SD)	Leukocyte Count, % (95% CI)	Leukopenia OR (95% CI)	Leukocytosis OR (95% CI)	n
A	25(OH)D	−1.6 (−2.1 to −1.0)	0.97 (0.89–1.07)	0.89 (0.84–0.93)	20,525
	cTAC	+2.9 (+2.3 to +3.5)	0.74 (0.66–0.82)	1.07 (1.01–1.13)	20,525
C	25(OH)D	−1.6 (−2.2 to −1.1)	1.09 (1.01–1.18)	0.92 (0.88–0.97)	20,525
	Albumin	+1.5 (+0.8 to +2.2)	0.60 (0.57–0.65)	0.84 (0.80–0.88)	20,525
	Urate	+3.5 (+2.8 to +4.1)	0.85 (0.76–0.94)	1.19 (1.13–1.25)	20,525
	Unconjugated bilirubin	−4.6 (−5.8 to −3.3)	1.19 (1.08–1.31)	0.71 (0.64–0.79)	20,525
	Direct bilirubin	+1.1 (−0.1 to +2.2)	0.96 (0.89–1.03)	1.09 (1.00–1.19)	20,525
D	25(OH)D	−1.2 (−1.8 to −0.6)	1.06 (0.98–1.16)	0.93 (0.87–1.00)	13,320
	Albumin	+0.8 (0.0 to +1.6)	0.60 (0.56–0.65)	0.76 (0.71–0.80)	13,320
	Urate	+4.2 (+3.2 to +5.2)	0.81 (0.71–0.92)	1.23 (1.14–1.32)	13,320
	Unconjugated bilirubin	−4.3 (−5.7 to −2.9)	1.16 (1.07–1.25)	0.71 (0.63–0.82)	13,320
	Direct bilirubin	+1.1 (−0.1 to +2.3)	0.95 (0.89–1.01)	1.07 (0.98–1.18)	13,320

Leukocyte count: percentage difference from linear models of log leukocyte count; leukopenia and leukocytosis: OR from logistic models, each compared with all other patient-years. Model A, 25(OH)D and cTAC; model C, 25(OH)D and components entered simultaneously; model D, model C plus log eGFR and log ALT. All models adjusted for age (B-spline, 4 df), sex, season and calendar era; 95% CI clustered by patient. Unconjugated bilirubin calculated as total minus direct bilirubin. SD: 25(OH)D 31.5 nmol/L; cTAC 128 µmol/L TE; albumin 4.9 g/L; urate 123.1 µmol/L; unconjugated bilirubin 7.7 µmol/L; direct bilirubin 10.9 µmol/L. 25(OH)D, 25-hydroxyvitamin D; ALT, alanine aminotransferase; CI, confidence interval; cTAC, calculated total antioxidant capacity; eGFR, estimated glomerular filtration rate; OR, odds ratio; SD, standard deviation; TE, Trolox equivalents.

**Table 3:** Association of urate and calculated unconjugated bilirubin with leukocyte outcomes after adjustment for haematological, hepatic and nutritional covariates, and in patient-years meeting the stated haematological restrictions (model D, per SD).

Model/Analysis Set	n	Urate, %	Urate, Leukocytosis OR	UCB, %	UCB, Leukocytosis OR	UCB, Leukopenia OR
Model D, reference (haemoglobin/platelet subset)	13,313	+4.2 (+3.2 to +5.2)	1.23 (1.14–1.32)	−4.3 (−5.7 to −2.9)	0.71 (0.63–0.81)	1.16 (1.07–1.25)
+ haemoglobin	13,313	+4.0 (+3.0 to +4.9)	1.22 (1.13–1.32)	−4.7 (−6.3 to −3.0)	0.69 (0.60–0.79)	1.16 (1.02–1.32)
+ log platelet count	13,313	+4.8 (+4.0 to +5.7)	1.26 (1.17–1.36)	−1.4 (−2.1 to −0.7)	0.93 (0.84–1.03)	0.96 (0.88–1.05)
+ haemoglobin and log platelet count	13,313	+4.6 (+3.8 to +5.5)	1.25 (1.16–1.35)	−1.8 (−2.7 to −0.8)	0.89 (0.79–1.00)	0.97 (0.90–1.04)
+ haemoglobin, log platelet count, MCV and RDW	13,311	+4.3 (+3.4 to +5.1)	1.22 (1.13–1.32)	−1.8 (−2.7 to −0.9)	0.88 (0.79–0.99)	0.96 (0.89–1.04)
Model D, reference (GGT/ALP subset)	12,239	+4.6 (+3.6 to +5.6)	1.30 (1.20–1.40)	−4.3 (−5.7 to −2.8)	0.69 (0.59–0.80)	1.15 (1.06–1.24)
+ log GGT and log ALP	12,239	+4.6 (+3.6 to +5.6)	1.28 (1.19–1.39)	−4.3 (−5.7 to −2.8)	0.69 (0.59–0.81)	1.15 (1.06–1.25)
Model D, reference (protein/glucose subset)	12,798	+4.1 (+3.1 to +5.2)	1.23 (1.14–1.33)	−4.4 (−5.9 to −2.9)	0.72 (0.63–0.82)	1.15 (1.07–1.24)
+ total protein and glucose	12,798	+4.3 (+3.3 to +5.3)	1.24 (1.15–1.34)	−4.4 (−5.8 to −2.9)	0.72 (0.63–0.83)	1.15 (1.07–1.24)
No anaemia	10,104	+4.9 (+3.9 to +6.0)	1.19 (1.07–1.32)	−5.4 (−6.6 to −4.1)	0.68 (0.59–0.79)	1.28 (1.06–1.55)
Platelets 150–400 × 10^9^/L and no anaemia	9094	+5.0 (+4.0 to +6.1)	1.19 (1.07–1.33)	−3.8 (−5.0 to −2.7)	0.76 (0.64–0.89)	1.06 (0.88–1.29)
+ haemoglobin and log platelet count	9094	+4.4 (+3.4 to +5.4)	1.15 (1.03–1.29)	−3.6 (−4.7 to −2.5)	0.80 (0.69–0.93)	1.09 (0.91–1.30)
RDW ≤ 15%	10,667	+4.5 (+3.4 to +5.6)	1.20 (1.09–1.32)	−4.5 (−6.8 to −2.2)	0.70 (0.53–0.91)	1.17 (1.01–1.36)

%, percentage difference in leukocyte count per SD; odds ratios per SD; 95% CI in parentheses. UCB, unconjugated bilirubin calculated as total minus direct bilirubin. All rows are model D (Table 2) with the additional covariates or restrictions listed; each reference row is model D refitted in the subset with the corresponding covariates available. Anaemia, haemoglobin < 120 g/L (women) or <130 g/L (men). GGT, γ-glutamyltransferase; ALP, alkaline phosphatase; MCV, mean corpuscular volume; RDW, red cell distribution width; CI, confidence interval; OR, odds ratio; SD, standard deviation.

**Table 4:** Sensitivity analyses of model D: percentage difference in leukocyte count (95% CI) per SD.

Analysis Set	n	Urate	Unconjugated Bilirubin	Albumin	25(OH)D
Primary analysis (patient-years)	13,320	+4.2 (+3.2 to +5.2)	−4.3 (−5.7 to −2.9)	+0.8 (0.0 to +1.6)	−1.2 (−1.8 to −0.6)
One record per patient (first eligible, 2014–2023)	9391	+3.3 (+2.3 to +4.4)	−4.7 (−5.8 to −3.5)	+1.1 (+0.2 to +2.0)	−1.1 (−1.8 to −0.4)
Excluding leukocytes ≥ 30 × 10^9^/L	13,314	+4.1 (+3.1 to +5.1)	−4.3 (−5.7 to −2.9)	+0.8 (0.0 to +1.6)	−1.2 (−1.8 to −0.6)
Excluding total bilirubin > 34 µmol/L	12,856	+4.9 (+3.9 to +5.9)	−2.4 (−4.0 to −0.8)	−1.0 (−1.8 to −0.1)	−1.2 (−1.8 to −0.6)
Excluding eGFR < 30 mL/min/1.73 m^2^	11,837	+2.7 (+1.6 to +3.8)	−4.2 (−5.6 to −2.8)	+1.0 (+0.1 to +1.9)	−1.2 (−1.9 to −0.5)
Excluding albumin < 35 g/L	12,607	+4.3 (+3.4 to +5.2)	−4.1 (−5.9 to −2.4)	+0.1 (−0.9 to +1.0)	−1.3 (−1.9 to −0.6)
Women	8234	+5.4 (+4.0 to +6.8)	−5.0 (−7.4 to −2.6)	−0.5 (−1.6 to +0.7)	−1.2 (−2.0 to −0.3)
Men	5086	+3.6 (+2.3 to +5.0)	−3.7 (−5.3 to −2.0)	+3.5 (+2.2 to +4.8)	−1.6 (−2.6 to −0.5)
2014–2019	4809	+5.5 (+4.0 to +7.0)	−5.5 (−7.0 to −4.0)	+2.5 (+1.1 to +3.9)	−1.4 (−2.7 to −0.2)
2020–2023	8511	+3.2 (+2.1 to +4.4)	−3.1 (−4.8 to −1.5)	−0.1 (−1.2 to +0.9)	−1.2 (−1.9 to −0.5)
Model D without ALT (eGFR only)	20,508	+4.3 (+3.5 to +5.0)	−4.9 (−6.3 to −3.5)	+0.9 (+0.2 to +1.6)	−1.3 (−1.9 to −0.8)
Multiple imputation of ALT and eGFR	20,525	+4.3 (+3.6 to +5.0)	−4.9 (−6.3 to −3.5)	+0.9 (+0.3 to +1.6)	−1.4 (−1.9 to −0.8)
Calendar-year fixed effects	13,320	+4.1 (+3.1 to +5.1)	−4.5 (−5.9 to −3.0)	+0.7 (−0.1 to +1.6)	−1.1 (−1.8 to −0.5)
Analytes standardised within calendar year	13,320	+3.9 (+2.9 to +4.8)	−4.2 (−5.5 to −2.9)	+0.5 (−0.3 to +1.4)	−1.2 (−1.8 to −0.5)
Patients with a single patient-year	7184	+2.8 (+1.6 to +4.0)	−4.6 (−6.0 to −3.2)	+1.0 (0.0 to +2.0)	−1.2 (−1.9 to −0.4)
Patients with ≥2 patient-years	6136	+5.2 (+3.6 to +6.8)	−4.1 (−6.2 to −1.9)	+0.9 (−0.6 to +2.3)	−0.9 (−2.0 to +0.1)
Weighted by 1/number of patient-years	13,320	+3.4 (+2.4 to +4.4)	−4.4 (−5.6 to −3.2)	+0.8 (0.0 to +1.7)	−1.1 (−1.7 to −0.5)
Leukocytes 4 to <10 × 10^9^/L (linear model)	11,140	+2.6 (+2.0 to +3.3)	−2.4 (−3.3 to −1.4)	+1.0 (+0.4 to +1.5)	−0.8 (−1.3 to −0.3)

Model D as in Table 2. Sex- and period-specific models omit the stratifying covariate. Multiple imputation: 20 imputations by chained equations, combined by Rubin’s rules. 25(OH)D, 25-hydroxyvitamin D; ALT, alanine aminotransferase; CI, confidence interval; eGFR, estimated glomerular filtration rate; SD, standard deviation.

## Data Availability

Patient-level data cannot be shared publicly because of privacy and ethics restrictions. Aggregated tables and the analysis code are available from the corresponding author subject to approval of the data controller.

## Supplementary Materials

The following supporting information can be downloaded at: https://www.mdpi.com/article/10.3390/ijms27219483/s1.

## References

[1] Sies H. (2015). Oxidative stress: A concept in redox biology and medicine. Redox Biol..

[2] Miller N.J., Rice-Evans C., Davies M.J., Gopinathan V., Milner A. (1993). A novel method for measuring antioxidant capacity and its application to monitoring the antioxidant status in premature neonates. Clin. Sci..

[3] Benzie I.F., Strain J.J. (1996). The ferric reducing ability of plasma (FRAP) as a measure of “antioxidant power”: The FRAP assay. Anal. Biochem..

[4] Rice-Evans C., Miller N.J. (1994). Total antioxidant status in plasma and body fluids. Methods in Enzymology.

[5] Miller N.J., Johnston J.D., Collis C.S., Rice-Evans C. (1997). Serum total antioxidant activity after myocardial infarction. Ann. Clin. Biochem. Int. J. Lab. Med..

[6] Bartosz G. (2010). Non-enzymatic antioxidant capacity assays: Limitations of use in biomedicine. Free Radic. Res..

[7] Prior R.L., Cao G. (1999). In vivo total antioxidant capacity: Comparison of different analytical methods. Free Radic. Biol. Med..

[8] Ames B.N., Cathcart R., Schwiers E., Hochstein P. (1981). Uric acid provides an antioxidant defense in humans against oxidant- and radical-caused aging and cancer: A hypothesis. Proc. Natl. Acad. Sci. USA.

[9] Sautin Y.Y., Johnson R.J. (2008). Uric acid: The oxidant-antioxidant paradox. Nucleosides Nucleotides Nucleic Acids.

[10] Berry C.E., Hare J.M. (2004). Xanthine oxidoreductase and cardiovascular disease: Molecular mechanisms and pathophysiological implications. J. Physiol..

[11] Martinon F., Pétrilli V., Mayor A., Tardivel A., Tschopp J. (2006). Gout-associated uric acid crystals activate the NALP3 inflammasome. Nature.

[12] Shi Y., Evans J.E., Rock K.L. (2003). Molecular identification of a danger signal that alerts the immune system to dying cells. Nature.

[13] Ruggiero C., Cherubini A., Ble A., Bos A.J., Maggio M., Dixit V.D., Lauretani F., Bandinelli S., Senin U., Ferrucci L. (2006). Uric acid and inflammatory markers. Eur. Heart J..

[14] Lyngdoh T., Marques-Vidal P., Paccaud F., Preisig M., Waeber G., Bochud M., Vollenweider P. (2011). Elevated serum uric acid is associated with high circulating inflammatory cytokines in the population-based Colaus study. PLoS ONE.

[15] Johnson R.J., Nakagawa T., Jalal D., Sánchez-Lozada L.G., Kang D.H., Ritz E. (2013). Uric acid and chronic kidney disease: Which is chasing which?. Nephrol. Dial. Transplant..

[16] Stocker R., Yamamoto Y., McDonagh A.F., Glazer A.N., Ames B.N. (1987). Bilirubin is an antioxidant of possible physiological importance. Science.

[17] Baranano D.E., Rao M., Ferris C.D., Snyder S.H. (2002). Biliverdin reductase: A major physiologic cytoprotectant. Proc. Natl. Acad. Sci. USA.

[18] Maghzal G.J., Leck M.C., Collinson E., Li C., Stocker R. (2009). Limited role for the bilirubin-biliverdin redox amplification cycle in the cellular antioxidant protection by biliverdin reductase. J. Biol. Chem..

[19] Keshavan P., Deem T.L., Schwemberger S.J., Babcock G.F., Cook-Mills J.M., Zucker S.D. (2005). Unconjugated bilirubin inhibits VCAM-1-mediated transendothelial leukocyte migration. J. Immunol..

[20] Lanone S., Bloc S., Foresti R., Almolki A., Taillé C., Callebert J., Conti M., Goven D., Aubier M., Dureuil B. (2005). Bilirubin decreases nos2 expression via inhibition of NAD(P)H oxidase: Implications for protection against endotoxic shock in rats. FASEB J..

[21] Wegiel B., Nemeth Z., Correa-Costa M., Bulmer A.C., Otterbein L.E. (2014). Heme oxygenase-1: A metabolic nike. Antioxid. Redox Signal..

[22] Wagner K.H., Wallner M., Mölzer C., Gazzin S., Bulmer A.C., Tiribelli C., Vitek L. (2015). Looking to the horizon: The role of bilirubin in the development and prevention of age-related chronic diseases. Clin. Sci..

[23] Vítek L., Jirsa M., Brodanová M., Kalab M., Marecek Z., Danzig V., Novotný L., Kotal P. (2002). Gilbert syndrome and ischemic heart disease: A protective effect of elevated bilirubin levels. Atherosclerosis.

[24] Tsai W.N., Wang Y.Y., Liang J.T., Lin S.Y., Sheu W.H., Chang W.D. (2015). Serum total bilirubin concentrations are inversely associated with total white blood cell counts in an adult population. Ann. Clin. Biochem. Int. J. Lab. Med..

[25] Hwang H.J., Lee S.W., Kim S.H. (2011). Relationship between bilirubin and C-reactive protein. Clin. Chem. Lab. Med..

[26] Roche M., Rondeau P., Singh N.R., Tarnus E., Bourdon E. (2008). The antioxidant properties of serum albumin. FEBS Lett..

[27] Gabay C., Kushner I. (1999). Acute-phase proteins and other systemic responses to inflammation. N. Engl. J. Med..

[28] Berridge M.J. (2015). Vitamin D cell signalling in health and disease. Biochem. Biophys. Res. Commun..

[29] Nakai K., Fujii H., Kono K., Goto S., Kitazawa R., Kitazawa S., Hirata M., Shinohara M., Fukagawa M., Nishi S. (2014). Vitamin D activates the Nrf2-Keap1 antioxidant pathway and ameliorates nephropathy in diabetic rats. Am. J. Hypertens..

[30] Charoenngam N., Holick M.F. (2020). Immunologic effects of vitamin D on human health and disease. Nutrients.

[31] Winterbourn C.C., Kettle A.J., Hampton M.B. (2016). Reactive oxygen species and neutrophil function. Annu. Rev. Biochem..

[32] Madjid M., Awan I., Willerson J.T., Casscells S.W. (2004). Leukocyte count and coronary heart disease: Implications for risk assessment. J. Am. Coll. Cardiol..

[33] Hernández-Ruiz Á., García-Villanova B., Guerra-Hernández E., Amiano P., Ruiz-Canela M., Molina-Montes E. (2019). A review of a priori defined oxidative balance scores relative to their components and impact on health outcomes. Nutrients.

[34] Howard S.C., Jones D.P., Pui C.H. (2011). The tumor lysis syndrome. N. Engl. J. Med..

[35] Crişan T.O., Cleophas M.C.P., Oosting M., Lemmers H., Toenhake-Dijkstra H., Netea M.G., Jansen T.L., Joosten L.A.B. (2016). Soluble uric acid primes TLR-induced proinflammatory cytokine production by human primary cells via inhibition of IL-1Ra. Ann. Rheum. Dis..

[36] Braga T.T., Forni M.F., Correa-Costa M., Ramos R.N., Barbuto J.A., Branco P., Castoldi A., Hiyane M.I., Davanso M.R., Latz E. (2017). Soluble uric acid activates the NLRP3 inflammasome. Sci. Rep..

[37] Qamar A.A., Grace N.D., Groszmann R.J., Garcia-Tsao G., Bosch J., Burroughs A.K., Ripoll C., Maurer R., Planas R., Escorsell A. (2009). Incidence, prevalence, and clinical significance of abnormal hematologic indices in compensated cirrhosis. Clin. Gastroenterol. Hepatol..

[38] Doumas B.T., Wu T.W. (1991). The measurement of bilirubin fractions in serum. Crit. Rev. Clin. Lab. Sci..

[39] Hutcheon J.A., Chiolero A., Hanley J.A. (2010). Random measurement error and regression dilution bias. BMJ.

[40] Soeters P.B., Wolfe R.R., Shenkin A. (2019). Hypoalbuminemia: Pathogenesis and clinical significance. JPEN J. Parenter. Enter. Nutr..

[41] VanderWeele T.J., Ding P. (2017). Sensitivity analysis in observational research: Introducing the E-value. Ann. Intern. Med..

[42] George J., Carr E., Davies J., Belch J.J.F., Struthers A. (2006). High-dose allopurinol improves endothelial function by profoundly reducing vascular oxidative stress and not by lowering uric acid. Circulation.

[43] Nakagawa M., Terashima T., D’yachkova Y., Bondy G.P., Hogg J.C., van Eeden S.F. (1998). Glucocorticoid-induced granulocytosis: Contribution of marrow release and demargination of intravascular granulocytes. Circulation.

[44] Osredkar J., Godnov U., Homšak Šturm E., Jerin A., Žiberna L. (2026). Serum 25-hydroxyvitamin D and serum urate in a selected tertiary-laboratory request stream: A retrospective cross-sectional study, 2014–2023. Biochem. Med..

[45] von Elm E., Altman D.G., Egger M., Pocock S.J., Gøtzsche P.C., Vandenbroucke J.P. (2007). The Strengthening the Reporting of Observational Studies in Epidemiology (STROBE) statement: Guidelines for reporting observational studies. Lancet.

[46] White I.R., Royston P., Wood A.M. (2011). Multiple imputation using chained equations: Issues and guidance for practice. Stat. Med..

