# Supplementary material for: Calculated Serum Antioxidant Capacity Masks Opposite Associations of Urate and Unconjugated Bilirubin with Leukocyte Count: A Ten-Year Cross-Sectional Hospital Laboratory Study Including 25-Hydroxyvitamin D
Source: Int J Mol Sci. 2026 Oct 23;27(21):9483. doi: 10.3390/ijms27219483 (PMC13762765; doi:10.3390/ijms27219483)
Supplement: Supplementary file 1 [file ijms-27-09483-s001.zip › ijms-4635551-supplementary.pdf]

International Journal of
Molecular Sciences

MDPI

Supplementary Materials

Calculated Serum Antioxidant Capacity Masks Opposite Associations of Urate and Unconjugated Bilirubin with Leukocyte Count: A Ten-Year Cross-Sectional Hospital Laboratory Study Including 25-Hydroxyvitamin D

Table S1. STROBE flow of laboratory records (tabular form).

Step	Records, n
All laboratory records 2014–2023	107,900
With a valid serum 25(OH)D result	107,856
With valid date, sex and date of birth	107,856
Adults aged 18–100 years	82,235
With all index analytes co-measured and plausible	24,811
PRIMARY COHORT: first record per patient per calendar year	20,527

After exclusion of records with missing base covariates, the analytic cohort comprised 20,525 patient-years from 14,152 individuals.

Table S2. Contribution of each component to the level (% of cTAC; P25, median, P75) and to the variance of calculated total antioxidant capacity.

Component	P25	Median	P75	Share of cTAC variance, %	r with cTAC
Albumin	46.4	52.5	58.7	7.1	0.19
Urate	38.7	45.2	51.6	90.5	0.92
Bilirubin	1.3	1.9	2.6	2.4	0.12

Share of variance = cov(component term, cTAC)/var(cTAC) across analytic patient-years; shares sum to 100%. The decomposition is descriptive and does not separate within-patient from between-patient variation. r, Pearson correlation of the component term with cTAC. cTAC, calculated total antioxidant capacity.

Table S3. Spearman correlations with leukocyte count and 25(OH)D.

Variable	ϱ with leukocyte count	ϱ with 25(OH)D
25(OH)D	−0.048	—
cTAC (composite)	0.082	−0.034
cTAC without albumin	0.073	−0.099
Albumin	0.012	0.167
Urate	0.092	−0.095
Unconjugated bilirubin (calculated)	−0.145	0.073
Direct bilirubin	−0.163	0.034

25(OH)D, 25-hydroxyvitamin D; cTAC, calculated total antioxidant capacity.

Table S4. Leukocyte outcomes by quintile of cTAC.

cTAC quintile	Range, µmol/L TE	n	Median leukocytes, $\times 10^9$/L	Leukopenia, %	Leukocytosis, %
Q1	326–689	4,105	6.9	5.7	15.2
Q2	689–755	4,105	6.7	4.7	10.6
Q3	755–818	4,106	6.9	3.6	10.0
Q4	818–896	4,104	7.1	2.0	12.0
Q5	897–1906	4,105	7.4	3.1	14.2

cTAC, calculated total antioxidant capacity; TE, Trolox equivalents.

Table S5a. Full estimates of models A–F, per SD of each predictor.

Model	Predictor (per SD)	Leukocyte count, % (95% CI)	Leukopenia OR (95% CI)	Leukocytosis OR (95% CI)	n
A	25(OH)D	−1.6 (−2.1 to −1.0)	0.97 (0.89–1.07)	0.89 (0.84–0.93)	20,525
	cTAC	+2.9 (+2.3 to +3.5)	0.74 (0.66–0.82)	1.07 (1.01–1.13)	20,525
B	25(OH)D	−1.6 (−2.1 to −1.0)	0.97 (0.88–1.08)	0.89 (0.84–0.93)	20,525
	cTAC	+2.9 (+2.3 to +3.5)	0.74 (0.66–0.83)	1.07 (1.01–1.13)	20,525
	25(OH)D × cTAC	−0.1 (−0.6 to +0.5)	1.01 (0.86–1.17)	1.01 (0.96–1.06)	20,525
C	25(OH)D	−1.6 (−2.2 to −1.1)	1.09 (1.01–1.18)	0.92 (0.88–0.97)	20,525
	Albumin	+1.5 (+0.8 to +2.2)	0.60 (0.57–0.65)	0.84 (0.80–0.88)	20,525
	Urate	+3.5 (+2.8 to +4.1)	0.85 (0.76–0.94)	1.19 (1.13–1.25)	20,525
	Unconjugated bilirubin	−4.6 (−5.8 to −3.3)	1.19 (1.08–1.31)	0.71 (0.64–0.79)	20,525
	Direct bilirubin	+1.1 (−0.1 to +2.2)	0.96 (0.89–1.03)	1.09 (1.00–1.19)	20,525
D	25(OH)D	−1.2 (−1.8 to −0.6)	1.06 (0.98–1.16)	0.93 (0.87–1.00)	13,320
	Albumin	+0.8 (0.0 to +1.6)	0.60 (0.56–0.65)	0.76 (0.71–0.80)	13,320
	Urate	+4.2 (+3.2 to +5.2)	0.81 (0.71–0.92)	1.23 (1.14–1.32)	13,320
	Unconjugated bilirubin	−4.3 (−5.7 to −2.9)	1.16 (1.07–1.25)	0.71 (0.63–0.82)	13,320
	Direct bilirubin	+1.1 (−0.1 to +2.3)	0.95 (0.89–1.01)	1.07 (0.98–1.18)	13,320
	log eGFR	+3.9 (+2.2 to +5.5)	0.89 (0.74–1.06)	1.28 (1.16–1.43)	13,320
	log ALT	−0.3 (−1.6 to +1.0)	1.12 (0.96–1.30)	0.99 (0.89–1.11)	13,320
E	25(OH)D	−1.3 (−2.0 to −0.7)	1.06 (0.97–1.16)	0.93 (0.87–0.99)	13,320
	Non-albumin cTAC	+2.6 (+1.7 to +3.6)	0.90 (0.80–1.01)	1.14 (1.06–1.23)	13,320
	25(OH)D × non-albumin cTAC	+0.4 (−0.3 to +1.1)	0.95 (0.88–1.03)	1.03 (0.97–1.09)	13,320
	Albumin	+1.2 (+0.3 to +2.0)	0.59 (0.55–0.63)	0.77 (0.73–0.81)	13,320
	log eGFR	+2.1 (+0.5 to +3.7)	0.97 (0.81–1.16)	1.15 (1.05–1.27)	13,320
	log ALT	−1.0 (−2.3 to +0.3)	1.16 (1.01–1.34)	0.95 (0.85–1.07)	13,320
F	25(OH)D	−1.1 (−1.8 to −0.5)	1.05 (0.95–1.15)	0.92 (0.86–0.99)	13,320
	Albumin	+0.8 (−0.1 to +1.6)	0.61 (0.56–0.65)	0.76 (0.71–0.81)	13,320
	Urate	+4.2 (+3.2 to +5.2)	0.80 (0.70–0.91)	1.23 (1.14–1.33)	13,320
	Unconjugated bilirubin	−4.3 (−5.7 to −2.9)	1.16 (1.08–1.24)	0.70 (0.62–0.79)	13,320
	Direct bilirubin	+1.1 (0.0 to +2.1)	0.94 (0.88–1.01)	1.05 (0.97–1.14)	13,320
	25(OH)D × urate	+0.3 (−0.3 to +1.0)	0.94 (0.86–1.04)	1.03 (0.97–1.09)	13,320
	25(OH)D × unconjugated bilirubin	0.0 (−1.2 to +1.1)	0.99 (0.94–1.04)	0.91 (0.80–1.02)	13,320
	log eGFR	+3.9 (+2.2 to +5.6)	0.88 (0.74–1.06)	1.29 (1.17–1.44)	13,320
	log ALT	−0.3 (−1.6 to +1.0)	1.12 (0.96–1.30)	1.00 (0.89–1.12)	13,320

Model B adds the 25(OH)D × cTAC interaction to model A; model E, non-albumin cTAC with its 25(OH)D interaction, albumin, log eGFR and log ALT; model F, model D plus 25(OH)D × urate and 25(OH)D × unconjugated bilirubin. Estimates for log eGFR and log ALT are per one-unit increase in the natural logarithm, not per SD. 25(OH)D, 25-hydroxyvitamin D; ALT, alanine aminotransferase; CI, confidence interval; cTAC, calculated total antioxidant capacity; eGFR, estimated glomerular filtration rate; OR, odds ratio; SD, standard deviation.

Table S5b. Supplementary checks: model C in the ALT/eGFR subset, model D with additional adjustment for haemoglobin and log platelet count, and model D restricted to direct bilirubin > 2 μmol/L.

Model	Predictor (per SD)	Leukocyte count, % (95% CI)	Leukopenia OR (95% CI)	Leukocytosis OR (95% CI)	n
C, ALT/eGFR subset	25(OH)D	−1.7 (−2.3 to −1.0)	1.08 (0.99–1.18)	0.91 (0.85–0.97)	13,320
	Albumin	+1.5 (+0.6 to +2.3)	0.59 (0.55–0.64)	0.79 (0.74–0.83)	13,320
	Urate	+3.1 (+2.2 to +4.1)	0.84 (0.74–0.95)	1.15 (1.07–1.23)	13,320
	Unconjugated bilirubin	−3.9 (−5.2 to −2.7)	1.15 (1.07–1.24)	0.76 (0.67–0.85)	13,320
	Direct bilirubin	+1.0 (−0.1 to +2.1)	0.96 (0.90–1.02)	1.07 (0.98–1.16)	13,320
D + haemoglobin, platelets	25(OH)D	−1.5 (−2.1 to −0.9)	1.12 (1.03–1.23)	0.92 (0.86–0.99)	13,313
	Albumin	−4.4 (−5.2 to −3.7)	1.11 (0.98–1.24)	0.63 (0.59–0.68)	13,313
	Urate	+4.6 (+3.8 to +5.5)	0.67 (0.59–0.77)	1.25 (1.16–1.35)	13,313
	Unconjugated bilirubin	−1.8 (−2.7 to −0.8)	0.97 (0.90–1.04)	0.89 (0.79–1.00)	13,313
	Direct bilirubin	+1.5 (+0.6 to +2.3)	0.95 (0.88–1.03)	1.08 (1.00–1.16)	13,313
D, direct bilirubin > 2 μmol/L	25(OH)D	−1.3 (−2.1 to −0.5)	1.06 (0.95–1.18)	0.94 (0.86–1.04)	8,857
	Albumin	+2.7 (+1.7 to +3.8)	0.58 (0.53–0.63)	0.80 (0.74–0.86)	8,857
	Urate	+3.8 (+2.6 to +4.9)	0.80 (0.70–0.93)	1.20 (1.10–1.32)	8,857
	Unconjugated bilirubin	−3.2 (−4.4 to −2.0)	1.12 (1.05–1.19)	0.82 (0.72–0.93)	8,857
	Direct bilirubin	+1.2 (+0.1 to +2.2)	0.95 (0.90–1.00)	1.07 (0.99–1.15)	8,857

25(OH)D, 25-hydroxyvitamin D; ALT, alanine aminotransferase; CI, confidence interval; eGFR, estimated glomerular filtration rate; OR, odds ratio; SD, standard deviation.

Table S6. STROBE checklist for cross-sectional studies.

Item	No.	Recommendation (cross-sectional studies)	Reported in
Title and abstract	1	(a) Indicate the study's design with a commonly used term in the title or the abstract; (b) provide an informative and balanced summary	Title; Abstract
Background/rationale	2	Explain the scientific background and rationale	Section 1
Objectives	3	State specific objectives, including any prespecified hypotheses	Section 1, last paragraph
Study design	4	Present key elements of study design early in the paper	Section 4.1
Setting	5	Describe the setting, locations and relevant dates	Section 4.1
Participants	6	Give the eligibility criteria and the sources and methods of selection	Section 4.2; Figure S1
Variables	7	Define all outcomes, exposures, potential confounders and effect modifiers	Sections 4.3–4.5
Data sources/measurement	8	Give sources of data and details of methods of assessment	Section 4.3
Bias	9	Describe any efforts to address potential sources of bias	Sections 4.2, 4.5, 2.5–2.8
Study size	10	Explain how the study size was arrived at	Section 2.1; Figure S1
Quantitative variables	11	Explain how quantitative variables were handled in the analyses	Sections 4.3–4.5
Statistical methods	12	(a) Statistical methods, including control for confounding; (b) subgroups and interactions; (c) missing data; (d) sampling strategy; (e) sensitivity analyses	Section 4.5; Sections 2.1, 2.5–2.8; Tables S6–S8
Participants	13	Report numbers of individuals at each stage of the study	Section 2.1; Figure S1
Descriptive data	14	Give characteristics of participants and indicate the number with missing data for each variable	Section 2.1; Table 1; Table S7
Outcome data	15	Report numbers of outcome events or summary measures	Section 2.1; Table 1; Table S3

Main results	16	Give unadjusted and confounder-adjusted estimates with their precision; report category boundaries	Sections 2.2–2.7; Tables 2 and 3; Figures 1–3; Tables S1–S4a
Other analyses	17	Report other analyses done, e.g., subgroups, interactions and sensitivity analyses	Sections 2.5–2.8; Tables 3 and 4; Tables S4b, S7–S10
Key results	18	Summarise key results with reference to study objectives	Section 3, first paragraph
Limitations	19	Discuss limitations, including sources of potential bias or imprecision	Sections 3.2, 3.5
Interpretation	20	Give a cautious overall interpretation of results	Sections 3.1–3.4
Generalisability	21	Discuss the generalisability (external validity) of the study results	Section 3.5
Funding	22	Give the source of funding and the role of the funders	Funding statement

Locations refer to section, table and figure numbers of the main manuscript (S, Supplementary Materials).

Table S7a. Model specifications.

Model	Exposure terms (per SD unless stated)	Adjustment	Sample (patient-years)
A	25(OH)D, cTAC	Base covariates	All patient-years (20,525)
B	Model A + 25(OH)D × cTAC	Base covariates	All patient-years (20,525)
C	25(OH)D, albumin, urate, calculated unconjugated and direct bilirubin	Base covariates	All patient-years (20,525)
D	As model C	Base covariates, log eGFR, log ALT	With eGFR and ALT (13,320)
E	25(OH)D, non-albumin cTAC, their interaction, and albumin	Base covariates, log eGFR, log ALT	With eGFR and ALT (13,320)
F	Model D + 25(OH)D × urate + 25(OH)D × unconjugated bilirubin	Base covariates, log eGFR, log ALT	With eGFR and ALT (13,320)
Splines (Figure 3)	B-spline (4 df) of cTAC, urate or unconjugated bilirubin; urate and bilirubin models adjusted for the other components	Base covariates, 25(OH)D	All patient-years (20,525)

Models A–F were fitted for three outcomes: log leukocyte count (ordinary least squares; coefficients back-transformed to % difference), leukopenia and leukocytosis (logistic regression, each versus all other patient-years); Figure 3 shows spline logistic models for the two binary outcomes. All splines: cubic B-splines with 4 df (patsy bs(); one internal knot at the median; boundary knots at the observed range; no intercept column). Base covariates: B-spline of age, sex, meteorological season (4 levels) and calendar era (2014–2019, 2020–2023). Exposures standardized over all patient-years; log eGFR and log ALT unstandardized. Standard errors cluster-robust by patient. Software: Python 3.13, statsmodels formula interface (patsy bs()). 25(OH)D, 25-hydroxyvitamin D; ALT, alanine aminotransferase; cTAC, calculated total antioxidant capacity; eGFR, estimated glomerular filtration rate; SD, standard deviation.

Table S7b. Physiological plausibility ranges.

Analyte	Unit	Plausibility range	Use
25(OH)D	nmol/L	1–500	Index analyte (record excluded if missing)
Leukocyte count	×10⁹/L	0.5–200	Index analyte (record excluded if missing)
Albumin	g/L	10–60	Index analyte (record excluded if missing)
Urate	µmol/L	30–1500	Index analyte (record excluded if missing)
Total bilirubin	µmol/L	1–800	Index analyte (record excluded if missing)
Direct bilirubin	µmol/L	0.5–700	Index analyte (record excluded if missing)
MCV	fL	50–140	Additional covariate (revision; value set to missing)
RDW	%	8–40	Additional covariate (revision; value set to missing)
GGT	µkat/L	0.02–200	Additional covariate (revision; value set to missing)

ALP	μkat/L	0.05–60	Additional covariate (revision; value set to missing)
Total protein	g/L	25–120	Additional covariate (revision; value set to missing)
Glucose	mmol/L	1–60	Additional covariate (revision; value set to missing)

Values outside the range were set to missing. Creatinine, ALT, haemoglobin and platelet count were used as reported. MCV, mean corpuscular volume; RDW, red cell distribution width; GGT, γ-glutamyltransferase; ALP, alkaline phosphatase.

Table S8a. Missing covariate data.

**Covariate**	**Missing, % of 20,525 patient-years**
eGFR (creatinine)	0.1
ALT	35.0
Haemoglobin	0.0
Platelets	0.0
MCV	0.0
RDW	0.0
GGT	35.1
ALP	6.2
Total protein	2.8
Glucose	0.8

eGFR from creatinine (CKD-EPI 2021). Index analytes had no missing values by design.

Table S8b. Characteristics of patient-years included in and excluded from model D (complete eGFR and ALT).

**Characteristic**	**Included in model D**	**Excluded (ALT or eGFR missing)**
n	13,320	7,205
Age, years	59.0	70.1
Women, %	61.8	47.1
Leukocytes, $\times 10^9$/L	6.9	7.3
Leukopenia, %	4.5	2.5
Leukocytosis, %	11.8	13.4
Urate, μmol/L	319	404
Unconjugated bilirubin, μmol/L	7	6
Albumin, g/L	44	43
25(OH)D, nmol/L	61	54
eGFR, mL/min/1.73 m^2	89.4	45.3
Haemoglobin, g/L	134	130
Period 2020–2023, %	63.9	28.0

Values are medians unless stated otherwise. 25(OH)D, 25-hydroxyvitamin D; ALT, alanine aminotransferase; eGFR, estimated glomerular filtration rate.

Table S8c. Model D without ALT and after multiple imputation of missing ALT and eGFR.

Model	Predictor	Leukocyte count, % (95% CI)	Leukopenia OR (95% CI)	Leukocytosis OR (95% CI)	n
D without ALT (eGFR only)	25(OH)D	−1.3 (−1.9 to −0.8)	1.08 (1.00–1.17)	0.94 (0.89–0.99)	20,508
	Albumin	+0.9 (+0.2 to +1.6)	0.62 (0.57–0.66)	0.81 (0.77–0.85)	20,508
	Urate	+4.3 (+3.5 to +5.0)	0.82 (0.74–0.92)	1.24 (1.18–1.31)	20,508
	Unconjugated bilirubin	−4.9 (−6.3 to −3.5)	1.20 (1.08–1.33)	0.67 (0.60–0.76)	20,508
	Direct bilirubin	+1.1 (−0.1 to +2.4)	0.96 (0.89–1.03)	1.10 (1.00–1.21)	20,508
D, multiple imputation (m = 20)	25(OH)D	−1.4 (−1.9 to −0.8)	1.08 (1.00–1.17)	0.94 (0.89–0.99)	20,525
	Albumin	+0.9 (+0.3 to +1.6)	0.61 (0.57–0.66)	0.81 (0.77–0.85)	20,525
	Urate	+4.3 (+3.6 to +5.0)	0.82 (0.74–0.91)	1.25 (1.18–1.32)	20,525
	Unconjugated bilirubin	−4.9 (−6.3 to −3.5)	1.20 (1.08–1.33)	0.67 (0.60–0.76)	20,525
	Direct bilirubin	+1.2 (−0.1 to +2.4)	0.95 (0.88–1.02)	1.10 (1.00–1.21)	20,525

Multiple imputation by chained equations (statsmodels MICEData; predictive mean matching with 10 donors; 5 burn-in cycles before the first and 2 cycles between subsequent imputations; 20 imputed data sets; NumPy random seed 20261018). Imputation model: log leukocyte count, standardized analytes, log eGFR, log ALT, log AST, log GGT, haemoglobin, log platelet count, age (linear), sex, season and era; clustering of patient-years within patients was not modelled. Each data set was analysed with model D and patient-clustered standard errors; estimates combined by Rubin's rules. Missing auxiliary values were imputed within the same chained-equations procedure. No convergence diagnostics are reported; this analysis is exploratory, with linear age and no modelling of patient clustering during imputation. 25(OH)D, 25-hydroxyvitamin D; ALT, alanine aminotransferase; CI, confidence interval; eGFR, estimated glomerular filtration rate; OR, odds ratio.

Table S9a. Model D in clinical units.

Model	Predictor	Leukocyte count, % (95% CI)	Leukopenia OR (95% CI)	Leukocytosis OR (95% CI)	n
D, clinical units	25(OH)D, per 25 nmol/L	−1.0 (−1.5 to −0.5)	1.05 (0.98–1.13)	0.95 (0.90–1.00)	13,320
	Albumin, per 5 g/L	+0.8 (0.0 to +1.7)	0.60 (0.55–0.64)	0.75 (0.71–0.80)	13,320
	Urate, per 100 μmol/L	+3.4 (+2.6 to +4.2)	0.84 (0.76–0.93)	1.18 (1.11–1.25)	13,320
	Unconjugated bilirubin, per 5 μmol/L	−2.8 (−3.8 to −1.9)	1.10 (1.05–1.16)	0.80 (0.74–0.87)	13,320
	Direct bilirubin, per 5 μmol/L	+0.5 (0.0 to +1.1)	0.98 (0.95–1.00)	1.03 (0.99–1.08)	13,320

Same model as model D (Table 2), with predictors per the stated unit instead of per SD. 25(OH)D, 25-hydroxyvitamin D; CI, confidence interval; OR, odds ratio; SD, standard deviation.

Table S9b. Multinomial logistic model D with normal leukocyte count (4 to <10 × 10^9/L) as the reference category.

Contrast	Predictor (per SD)	Relative risk ratio (95% CI)	n
leukopenia vs. normal count	25(OH)D	1.06 (0.97–1.15)	13,320
	Albumin	0.57 (0.53–0.62)	13,320
	Urate	0.83 (0.73–0.94)	13,320
	Unconjugated bilirubin	1.14 (1.06–1.22)	13,320
	Direct bilirubin	0.95 (0.90–1.01)	13,320
leukocytosis vs. normal count	25(OH)D	0.93 (0.87–1.00)	13,320
	Albumin	0.72 (0.68–0.77)	13,320
	Urate	1.21 (1.12–1.31)	13,320
	Unconjugated bilirubin	0.72 (0.63–0.83)	13,320

	Direct bilirubin	1.08 (0.98–1.18)	13,320

Covariates as in model D; patient-clustered 95% CI. 25(OH)D, 25-hydroxyvitamin D; CI, confidence interval; SD, standard deviation.

Table S9c. Model D with alternative functional forms for age.

Model	Predictor	Leukocyte count, % (95% CI)	Leukopenia OR (95% CI)	Leukocytosis OR (95% CI)	n
D, age B-spline 3 df	Urate	+4.1 (+3.1 to +5.0)	0.82 (0.72–0.93)	1.22 (1.13–1.31)	13,320
	Unconjugated bilirubin	−4.4 (−5.8 to −3.0)	1.17 (1.08–1.26)	0.71 (0.62–0.81)	13,320
D, age B-spline 6 df	Urate	+4.1 (+3.1 to +5.1)	0.81 (0.71–0.92)	1.22 (1.14–1.32)	13,320
	Unconjugated bilirubin	−4.3 (−5.7 to −2.8)	1.16 (1.07–1.25)	0.71 (0.63–0.81)	13,320
D, linear age	Urate	+4.3 (+3.3 to +5.3)	0.81 (0.72–0.92)	1.23 (1.14–1.32)	13,320
	Unconjugated bilirubin	−4.4 (−5.8 to −2.9)	1.17 (1.07–1.27)	0.71 (0.62–0.81)	13,320

Primary specification: B-spline of age with 4 df (Table 2). CI, confidence interval; OR, odds ratio.

Table S9d. Median analyte concentrations by calendar year.

Year	Patient-years	Urate, μmol/L	Unconjugated bilirubin, μmol/L	Direct bilirubin, μmol/L	Albumin, g/L	Leukocytes, $\times 10^9$/L	25(OH)D, nmol/L
2014	1,417	392	6	3	42	6.9	50
2015	1,553	390	7	3	43	7	50
2016	1,947	397	6	3	43	6.9	50
2017	2,179	388	6	3	43	6.8	51
2018	1,282	340	7	3	43	7	54
2019	1,615	343	7	3	44	7	53
2020	1,839	319	7	3	44	7.3	64
2021	2,729	316	7	3	45	7.2	65
2022	2,675	334	7	3	44	7.1	66
2023	3,289	319	7	4	45	6.8	66

Changes over time may reflect case mix as well as analytical changes; calendar-year fixed effects and within-year standardization are shown in Table 4. 25(OH)D, 25-hydroxyvitamin D.

Table S10a. Alternative weighting of the calculated antioxidant index.

Weighting scheme	Weights (albumin, urate, bilirubin)	Urate / albumin / bilirubin share of variance, %	r with urate	Leukocyte count, % per SD (95% CI)	Leukopenia OR (95% CI)	Leukocytosis OR (95% CI)
TEAC coefficients (Miller et al.; main analysis)	0.63, 1.02, 1.5	90.5 / 7.1 / 2.4	0.92	+2.9 (+2.3 to +3.5)	0.74 (0.66–0.82)	1.07 (1.01–1.13)
TEAC, calculated unconjugated instead of total bilirubin	0.63, 1.02, 1.5	91.6 / 7.9 / 0.5	0.93	+3.3 (+2.6 to +3.9)	0.68 (0.61–0.76)	1.08 (1.02–1.13)
FRAP stoichiometry (albumin ~0)	0, 2, 4	93.9 / 0.0 / 6.1	0.96	+2.3 (+1.6 to +3.0)	1.02 (0.92–1.13)	1.16 (1.11–1.23)
Molar sum (all 1)	1, 1, 1	77.4 / 22.2 / 0.4	0.84	+3.1 (+2.5 to +3.7)	0.64 (0.59–0.71)	1.02 (0.96–1.07)
TEAC, albumin coefficient x0.5	0.315, 1.02, 1.5	96.8 / 0.3 / 2.9	0.97	+2.8 (+2.2 to +3.5)	0.84 (0.75–0.94)	1.12 (1.07–1.19)
TEAC, albumin coefficient x1.5	0.945, 1.02, 1.5	79.6 / 18.6 / 1.9	0.85	+2.9 (+2.3 to +3.5)	0.68 (0.61–0.74)	1.02 (0.97–1.08)
TEAC, urate coefficient x0.5	0.63, 0.51, 1.5	62.8 / 29.0 / 8.3	0.74	+2.1 (+1.5 to +2.7)	0.71 (0.65–0.78)	0.96 (0.92–1.02)
TEAC, urate coefficient x1.5	0.63, 1.53, 1.5	97.0 / 2.1 / 1.0	0.96	+3.2 (+2.5 to +3.8)	0.76 (0.68–0.85)	1.12 (1.06–1.18)
TEAC, bilirubin coefficient x0.5	0.63, 1.02, 0.75	92.2 / 7.6 / 0.2	0.93	+3.3 (+2.7 to +4.0)	0.69 (0.62–0.77)	1.08 (1.03–1.14)
TEAC, bilirubin coefficient x1.5	0.63, 1.02, 2.25	87.2 / 6.5 / 6.4	0.90	+2.5 (+1.8 to +3.1)	0.79 (0.71–0.88)	1.06 (1.00–1.11)

Each index was standardized and entered in place of cTAC in model A (n = 20,525). FRAP stoichiometric factors from Benzie and Strain (urate 2.0, bilirubin 4.0; albumin set to 0 because its FRAP activity is very low). Bilirubin term: total bilirubin unless stated.

Albumin in μmol/L (66.5 kDa). CI, confidence interval; cTAC, calculated total antioxidant capacity; OR, odds ratio; SD, standard deviation.

Table S10b. Variance in log leukocyte count explained by the covariates, cTAC, urate alone and the components, in the same samples.

Sample	Model of log leukocyte count	n	R^2, %	Adjusted R^2, %
All patient-years	Covariates only	20,525	0.76	0.71
	A: 25(OH)D + cTAC	20,525	1.62	1.57
	Urate alone	20,525	1.88	1.82
	C: components	20,525	3.57	3.51
ALT/eGFR subset	Covariates only	13,320	1.43	1.36
	A: 25(OH)D + cTAC	13,320	1.99	1.91
	Urate alone	13,320	2.22	2.13
	C: components	13,320	3.84	3.73
	Covariates + log eGFR + log ALT	13,320	1.56	1.48
	D: components + log eGFR + log ALT	13,320	4.32	4.21

Urate alone in place of cTAC (model A): leukocyte count +3.6% (+2.9 to +4.3) per SD versus +2.9% (+2.3 to +3.5) for cTAC; leukocytosis OR 1.21 (1.15–1.28) versus 1.07 (1.01–1.13). 25(OH)D, 25-hydroxyvitamin D; ALT, alanine aminotransferase; cTAC, calculated total antioxidant capacity; eGFR, estimated glomerular filtration rate; OR, odds ratio; SD, standard deviation.

Table S11a. Bilirubin measurement checks.

Model	Predictor	Leukocyte count, % (95% CI)	Leukopenia OR (95% CI)	Leukocytosis OR (95% CI)	n
D with total bilirubin at fixed direct bilirubin (measured analytes only)	Total bilirubin at fixed direct bilirubin, per SD of unconjugated bilirubin	−4.3 (−5.7 to −2.9)	1.16 (1.07–1.25)	0.71 (0.63–0.82)	13,320
D, direct bilirubin > 2	Urate	+3.8 (+2.6 to +4.9)	0.80 (0.70–0.93)	1.20 (1.10–1.32)	8,857
	Unconjugated bilirubin	−3.2 (−4.4 to −2.0)	1.12 (1.05–1.19)	0.82 (0.72–0.93)	8,857
D, direct bilirubin > 3	Urate	+3.2 (+1.8 to +4.6)	0.86 (0.74–1.00)	1.18 (1.06–1.32)	5,669
	Unconjugated bilirubin	−2.6 (−3.7 to −1.5)	1.09 (1.03–1.16)	0.84 (0.74–0.96)	5,669

Total and direct bilirubin were reported in whole μmol/L; direct bilirubin was ≤ 2 μmol/L in 36.8% and ≤ 3 μmol/L in 61.3% of patient-years; one record had total < direct bilirubin (unconjugated fraction set to 0). Because model D contains direct bilirubin, the estimate for total bilirubin at fixed direct bilirubin (both measured) equals that for the calculated unconjugated fraction; this is a reparameterization and does not validate measurement accuracy. CI, confidence interval; OR, odds ratio; SD, standard deviation.

Table S11b. Exploratory comparison of isolated unconjugated hyperbilirubinaemia (Gilbert-like phenotype) with normal bilirubin.

Model	Predictor	Leukocyte count, % (95% CI)	Leukopenia OR (95% CI)	Leukocytosis OR (95% CI)	n
Exploratory (n = 77 vs. 5,489)	Isolated unconjugated hyperbilirubinaemia vs. normal bilirubin	+0.3 (−6.2 to +7.2)	0.46 (0.06–3.57)	1.19 (0.63–2.24)	5,566

Isolated unconjugated hyperbilirubinaemia: total bilirubin > 17 μmol/L with ≥ 80% calculated unconjugated; reference: total bilirubin ≤ 17 μmol/L. Both groups required direct bilirubin ≤ 5 μmol/L and were restricted to patient-years without anaemia and with red cell distribution width ≤ 15%, ALT ≤ 35 U/L (women) or ≤ 45 U/L (men) and platelets 150–400 × 10^9/L. Eight otherwise eligible reference

patient-years lacked log eGFR, leaving 77 phenotype and 5,489 reference patient-years. Adjusted for base covariates, 25(OH)D, albumin, urate and log eGFR; patient-clustered 95% CI. Leukocytosis occurred in 10 and leukopenia in 1 of the 77 patient-years with isolated unconjugated hyperbilirubinaemia; estimates are imprecise. 25(OH)D, 25-hydroxyvitamin D; ALT, alanine aminotransferase; CI, confidence interval; eGFR, estimated glomerular filtration rate; OR, odds ratio.
